# Non-redundant functions of NFATc1 in survival and NFATc2 in generation of exhausted CD8^+^ T cells

**DOI:** 10.3389/fimmu.2026.1869029

**Published:** 2026-07-24

**Authors:** Salvador Sampere-Birlanga, Stefan Klein-Hessling, Miriam Campillo Prados, Anfei Huang, Hao Wu, Andreas Rosenwald, Wolfgang Kastenmüller, Edgar Serfling, Martin Vaeth, Friederike Berberich-Siebelt

**Affiliations:** 1 Department of Molecular Pathology, Institute of Pathology, Julius-Maximilians-University of Würzburg, Würzburg, Germany; 2 Würzburg Institute of Systems Immunology, Max Planck Research Group at the Julius-Maximilians University of Würzburg, Würzburg, Germany; 3 Institute of Pathology, Julius-Maximilians University of Würzburg, Würzburg, Germany; 4 Mildred Scheel Early Career Center (MSNZ) of the German Cancer Aid, University Hospital, Julius-Maximilians University of Würzburg, Würzburg, Germany

**Keywords:** CD8^+^ T cells, Nfatc1, NFATc2, T-cell exhaustion, T-cell survival

## Abstract

Persistent antigenic stimulation leads to the dysfunction of CD8^+^ cytotoxic T cells. These “exhausted” T_EX_ cells exhibit reduced proliferative capacity, impaired effector function, and increased expression of co-inhibitory receptors. Chronic antigen receptor stimulation induces the expression of NFATc1/αA, a short isoform of NFATc1 that promotes T_EX_ cell survival. The induction of NFATc1/αA is accompanied by a significant decrease in NFATc2 expression. NFATc2 limits the expression of stemness-associated genes, such as *Tcf7*, *Sell*, and *Id3.* It also supports the expression of *Prf1*, *Gzmb*, and the T_EX_ marker gene *Havcr2*. Therefore, ablation of NFATc2 mitigates functional exhaustion of CD8^+^ T cells during chronic viral infection and antitumor immunity. Our findings illustrate that NFATc1 promotes the survival of T_EX_ cells, while NFATc2 promotes the terminal differentiation and dysfunction of exhausted CD8^+^ T cells. The data suggest a non-redundant interplay between NFATc1 and NFATc2, each playing a distinct role in controlling CD8^+^ T-cell exhaustion. These findings open novel avenues to enhance the efficacy of immune checkpoint and CAR T-cell therapies.

## Introduction

Functional exhaustion of CD8^+^ T cells is a hallmark of chronic viral infections and anti-tumor immunity. Transcription factors (TF) of the family of *Nuclear Factor of Activated T-cells* (NFAT) are the most prominent downstream targets of the Ca^2+^/calcineurin (CN) signaling pathway in lymphocytes that control many aspects of T-cell fate, including T-cell activation, clonal expansion and exhaustion. Inhibiting CN phosphatase activity, and thereby the activity of NFATs, with the immune suppressants cyclosporin A (CsA) or tacrolimus (FK506) abolishes adaptive immune responses (1–4).

In peripheral lymphocytes, the two NFAT family members NFATc1 and NFATc2 are predominantly expressed. While both NFAT family members can bind to similar DNA motifs and seem to transactivate the promoters and enhancers of numerous genes in similar ways (1–3), inactivating the *Nfatc1* or *Nfatc2* genes in mice resulted in diverging phenotypes. Whereas inactivation of the *Nfatc1* gene leads to an early death of mouse embryos (5, 6), NFATc2*-*deficient mice are born at normal Mendelian ratios but develop an age-related hyper-proliferative syndrome of lymphocytes and elevated immune responses (7–9). By contrast, lymphocyte-specific ablation of NFATc1 in mice results in impaired T-cell expansion and effector function, which are not detected in mice bearing NFATc2-deficient lymphocytes (10, 11). These earlier data suggest that the individual NFAT factors differ remarkably in their function to control the immune system.

The differences between NFATc1 and NFATc2 are further supported by the strong induction of *Nfatc1* gene expression at the transcriptional level upon antigen receptor stimulation, whereas NFATc2 is constitutively expressed. Ca^2+^ signaling during lymphocyte activation rapidly induces the nuclear translocation of cytosolic NFAT proteins. Once activated, the *Nfatc1* gene – but not the *Nfatc2* gene – undergoes enhanced transcription through a positive autoregulatory mechanism, in which NFATc1 promotes its own expression (12). This property of *Nfatc1* auto-amplification prompted us to focus on the structure and expression of the *Nfatc1* gene. Due to two promoters, two polyA addition sites, and alternative splicing events, the *Nfatc1* gene gives rise to six prominent isoforms that are synthesized upon antigen receptor stimulation in primary murine and human lymphocytes (13, 14). However, robust primary or secondary stimulation of lymphocytes leads to the predominant expression of NFATc1/αA, a short isoform that differs in its transcriptional capacity and regulation from the longer NFATc1/B and C isoforms. In addition to a proximal polyA site (15), the induction of α-peptide-containing isoforms, predominantly NFATc1/αA, is directed by the inducible promoter P1, whose activity is supported by a remote enhancer, designated as E2. The E2 enhancer spans approximately 1.2 kb DNA and is situated within intron 10, the last intron of the *Nfatc1* gene (16). These findings, and the isoform-specific fine-tuning of NFATc1’s transcriptional activity by SUMOylation (17, 18), let us speculate that “two genes are located within the *Nfatc1* locus” (19).

Responses to both tumors and chronic viral infections result in the functional exhaustion of antigen-specific CD8^+^ T cells. These exhausted T (T_EX_) cells consist of multiple subsets that exhibit reduced proliferative capacity, increased expression of co-inhibitory receptors and resistance to checkpoint immune therapies (16–19). Contrary to the terminally exhausted T_EX_ cells that express at high levels the exhaustion markers PD-1, TIM-3, LAG-3, and TIGIT, progenitors of exhausted T cells (T_PEX_ cells) exhibit both low levels of exhaustion markers and a stem-cell like phenotype, including the expression of Ly108 and TCF-1 (20). Much research has been devoted to elucidating the molecular mechanisms that lead to T-cell exhaustion, and to prevent or even revert the exhausted phenotype.

Infections with “acute” and “chronic” strains of the lymphocytic choriomeningitis virus (LCMV) are well-established mouse models for studying CD8^+^ T-cell differentiation and functional exhaustion *in vivo* (21). Upon chronic infection with the “Docile” or “Clone 13” strains of LCMV, T cells express predominantly NFATc1/αA but low levels of NFATc2 proteins (22). Notably, evidence from three independent studies implicated CN/NFAT signaling as a critical regulator of TOX expression. TOX is a member of the HMG-box superfamily of TFs (23) that controls CD8^+^ T-cell exhaustion (24–26). TOX expression depends on CN signals (27), and when a constitutively active version of NFATc1 (in fact, the inducible NFATc1/αA isoform) was ectopically expressed in CD8^+^ T cells via retroviral transduction, TOX expression increased, whereas NFATc1-deficient T cells showed impaired TOX levels (24).

In addition to these findings, several other studies have suggested that NFAT proteins are key orchestrators of the transcriptional program underlying T-cell exhaustion (28). Previous studies on the role of NFATs in CD8^+^ T-cell differentiation led to the view that all NFAT family members, including NFATc1, can promote CD8^+^ T-cell exhaustion, when not interacting with Jun/Fos AP-1 factors (4). However, this notion seems to contradict the findings of our and other laboratories on specific functions of individual NFAT TFs in gene regulation, proliferation, and apoptosis of lymphocytes (29). For example, although acute MCMV infections can be controlled by NFATc1- and/or NFATc2-deficient T cells, NFATc1 is specifically necessary for the transition from central to effector memory, and for migration to non-lymphoid tissues (30). Furthermore, *Nfatc1*^–/–^ (or *Nfatc1*^–/–^*Nfatc2*^–/–^) CD8^+^ T cells differentiate less into terminally differentiated, migrating effector memory cells that control latent MCMV infections (30). However, the need for NFATc1 to control latent MCMV infections is reverted under strong inflammatory conditions, such as GvHD (31), demonstrating the context dependency. The hypothesis that both NFATc1 and NFATc2 control CD8^+^ T-cell exhaustion in different ways encouraged us to investigate the role of NFATc1 *versus* NFATc2 during the exhaustion of murine CD8^+^ T cells in greater detail. We show here that upon persistent antigenic stimulation, NFATc1/αA is strongly induced in CD8^+^ T cells at the transcriptional level. By contrast, NFATc2 protein was expressed at lower levels in exhausted than non-exhausted CD8^+^ T cells. NFATc1/αA supported the survival of exhausted CD8^+^ T cells. Ablation of NFATc1 led to upregulation of NFATc2, suggesting that NFATc1/αA prevents NFATc2 accumulation, as the ablation of NFATc2 enhanced NFATc1 protein levels. In addition, our data demonstrate that ablation of NFATc2 enhances the anti-tumor activity of CD8^+^ T cells and highlight its potential as a target in immunotherapeutic strategies, including CAR T-cell therapies.

## Materials and methods

### Mice

All mice were bred and maintained under specific pathogen free conditions in the Center for Experimental Medicine (ZEMM) or the Institute for Systems Immunology at the Julius-Maximilians-University of Würzburg: P14 (JAX strain 037394), Ubc-GFP (strain 004353), *Cd4-cre* (32), *Rag1*^–/–^ (strain 002216), OT-I (Tg TcraTcrb)^1100Mjb^, strain 003831), and tdTomato reporter mice (33). To make use of the congenic marker CD90.1, mice had been crossed to B6.L2G85.CD90.1 transgenic mice (34). The generation of *Nfatc1*^fl/fl^×*Cd4-cre* has been described (35, 36). The *Nfatc1*^fl/fl^.YFP.*Cd4-cre* mice were generated by crossing *Nfatc1*^fl/f^*^l^*×*Cd4-cre* with R26R.EYFP (strain 006148) mice. The latter carry the EYFP transgene preceded by a loxP-framed STOP codon inserted into the Rosa26 locus (37). To generate NFATc1c2-double-deficient mice, *Nfatc1*^fl/fl^×*Cd4cre* were inter-bred with *Nfatc2*^–/–^ mice (8, 35). In ΔE2 mice, *Cd4*-cre recognizes the two loxP sites flanking the remote enhancer E2 in intron 10 of the *Nfatc1* gene locus (38).

Ethical approval for all animal experiments was obtained from the appropriate authorities (*Regierung von Unterfranken*/Gov. Lower Franconia) and adhered to German animal protection regulations.

### Isolation of splenic T cells

Splenocytes were isolated, filtered through 0.70 µm nylon meshes, and centrifuged 400xg for 5 min. Erythrocyte lysis buffer (155 mM NH_4_Cl, 10 mM KHCO_3_, 0.1 mM EDTA; pH 7.2) was applied to lyse red blood cells. Alternatively, the gentle MACS™ dissociator from Miltenyi Biotech was used according to the manufacturer’s protocol. Positive selection of CD8^+^ T cells was followed using the CD8a (Ly-2) microbeads kit from Miltenyi Biotech. Isolated CD8^+^ T cells were seeded in a concentration of 0.5x10^6^ cells/ml in complete RPMI medium with IL-2 (100 Units/ml) in12-well plates.

### Cell culture

Plates were coated with αCD3 (3 µg/ml) and αCD28 (1 µg/ml) in 1–2 ml filtered PBS for 45–90 min (37 °C, 5% CO^2^). For primary stimulation, the cells were suspended in complete RPMI along with IL-2 (100 Units/ml). After 48h, the cells were removed from the wells, washed, and their number was determined. For “Acute stimulation” cells were kept in RPMI supplemented with IL-2 (100 Units/ml) only, while for “Chronic stimulation” IL-15 (15 ng/ml) and IL-7 (15 ng/ml) were utilized, and the cells were (at least 2x) stimulated by plate-bound αCD3. For chronic stimulation of OT-I cells, the protocol described by Zhao was followed (39).

### LCMV production and infection models

LCMV strains Armstong, Docile and Clone 13 viral stocks were produced using L929/BHK cells. Cells were infected with LCMV in a dose of 0.1 U/cell for 1h at RT, before the supernatant was aspirated and replaced. After 48h and 72h at 37 °C, the virus-containing supernatant was harvested. For the titration of viral stocks and the measurement of viral loads of organs, a limiting dilution assay was performed. 4x10^6^ cells/ml MC57G cells were seeded in 96-well plates and serial virus dilutions from 2x10–^2^ to 2x10–^6^ were added in replicates. The medium was exchanged after 48 and 72h incubation, cells were washed and permeabilized/fixed using Cytofix/Cytoperm kit (BD Biosciences). Cells were stained with VL-4 hybridoma cell line supernatant containing LCMV NP-specific antibodies for 60min at RT. As secondary antibody, an anti-rat IgG AF488 (Invitrogen) was used. Virus titers were calculated by counting wells with infected cells using an Axio Vert.A1 epifluorescence microscope (Zeiss). For LCMV-Armstrong infections, mice were infected intraperitoneally (i.p.) with an infectious dose of 2x10^5^ IU. LCMV-Docile and LCMV-Clone 13 virus stocks were diluted in sterile PBS before intravenous (i.v.) injection of an infectious dose of 4x10^6^ IU.

### Adoptive T-cell transfers before LCMV infection

Purified P14 CD8^+^ T cells were transferred i.v. in sterile PBS into recipient mice. In total 6x10^3^ P14 T cells were transferred 1d before LCMV infection. In competitive co-transfer experiments, GFP/tdTomato-, GFP- and tdTomato- expressing WT, NFATc1- and NFATc2-deficient P14 T cells were mixed in a 1:1:1 ratio (2x10^3^ each) before transfer.

### *In vivo* melanoma model and adoptive T-cell transfer

To generate melanoma tumors in *Rag1^-/-^* mice, 0.1 ml of “ECM-gel” matrigel (E127, Merck) was injected subcutaneously along with 0.1 ml of PBS containing 2x10^5^ B16.OVA.luci tumor cells. The mixture was allowed to sit for half a minute until the matrigel hardened. The mouse was then kept under daily observation for subsequent monitoring of tumor growth.

Concurrently, splenic CD8^+^ OT-I T cells were isolated, activated for 2d, and maintained under “acute” conditions with IL-2 (100 units/ml). After 6d, 5x10^6^ OT-I T cells were prepared in 0.2 ml PBS and kept on ice until injection into the tail vein of *Rag1^-/-^* mice, where the tumor size was approximately 0.3-0.4 cm². The mice were monitored daily, and tumor sizes were measured every two days until d12, when the mice were sacrificed by CO^2^ inhalation. Then, subcutaneous tumors were extracted using forceps and a scalpel, and the Tumour Dissociation Kit along with the gentleMACS™ Dissociator (both from Miltenyi Biotech) were used according to the protocol for the extraction of CD8^+^ Tumor Infiltrating Lymphocytes (TILs).

### Cytotoxicity assay

B16.OVA.Luci melanoma tumor cells were cultivated in complete DMEM (10% FCS), maintaining exponential cell growth. For cytotoxicity assays, IFN-γ was added to the B16.OVA.Luci melanoma cells for 15h to support MHC expression. Harvested cells were re-suspended at 10^6^ cells per well in complete RPMI supplemented with IFN-γ (10 ng/ml). 2h later, tumor cells had adhered to the bottom of the plate and 5 x 10^5^ T cells per well could be added at a 1:2 ratio. After further 8h, the Beetle-Juice Luciferase Assay Firefly Kit (PJK GmbH) was used according to the manufacturer’s protocol, and luminescence was measured using the LUMIstar Omega microplate reader to determine the cytotoxic capacity of the added CD8^+^ T cells.

### Flow cytometry

Flow cytometric staining (antibodies in **Table 1**) was performed as previously described (40). Briefly, cells were stained with Zombi NIR or Zombie Violet live/dead dye (eBioscience) for 10min in PBS at RT together with an anti-FcγRII/FcγRIII (anti-CD16/32) antibody (clone 2.4G2; Bio X Cell or Biolegend) to prevent unspecific binding. After washing, surface antigens were stained with fluorophore-conjugated antibodies in PBS containing 0.5% BSA for 20min at RT in the dark.

**Table 1:** Antibodies for flow cytometry.

Antibody name	Clone	Company/supplier
Cell Event Caspase 3/Detection Reagent		Thermo Fischer
Cleaved Caspase3 -Unconjugated-monocl.	5A1E	Cell Signaling
PI Annexin V Apoptosis Detection Kit		Biolegend
Anti-mouse Bcl-2	BCL/10C4	BD Biosciences
Anti-mouse BIM	C34C5	Cell Signaling
Anti-mouse CD44-APC	IM7	Biolegend
Anti-mouse CD44-AlexaFluor488	IM7	Biolegend
Anti-mouse CD44-BV510	IM7	Biolegend
Anti-mouse CD44-PerCP-Cy5.5	IM7	Biolegend
Anti-mouse CD62L-AmCyan	MEL-14	Biolegend
Anti-mouse CD69-PE	H1.2F3	Biolegend
Anti-mouse CD69-PerCP-Cy5.5	H1.2F3	Biolegend
Anti-mouse CD80-FITC	16-10A1	Biolegend
Anti-mouse CD8a-AmCyan	SK1	Biolegend
Anti-mouse CD8a-Brilliant Violet 510	SK1	Biolegend
Anti-mouse CD8a-AlexaFluor488	53-6.7	Biolegend
Anti-mouse CD8a-BV605	53-6.7	Biolegend
Anti-mouse CD8a-PE-Cy7	53-6.7	Biolegend
Anti-mouse CD90.1. (Thy1.1)-PE	KW322	Biolegend
Anti-mouse Eomes-PE	W17001A	Biolegend
Anti-mouse Eomes-PE-Cy7	Dan11mag	Thermo Fisher
Anti-mouse IFN-γ-PerCP-Cy5.5	XMG1.2	Biolegend
Anti-mouse IL-7Ra-PE-Cy7	A73R4	Biolegend
Anti-mouse Ki67- PerCP-Cy5.5	REA183	Miltenyi Biotech
Anti-mouse KLRG1-BUV661	2F1	BD Bioscience
Anti-mouse LAG-3-APC	C9B7W	Biolegend
Anti-mouse LAG3 BUV737	C9B7W	BD Bioscience
Anti-mouse Ly108-BV711	13G3	BD Bioscience
Anti-mouse Ly108(Slamf5)-PE	330-AJ	Biolegend
Anti-mouse PD-1-APC-Cy7	29F.1A12	Biolegend
Anti-mouse PD-1-BV605	29F.1A12	Biolegend
Anti-mouse Recombinant CD16/32	93	Biolegend
Anti-mouse T-bet-APC	4B10	Biolegend
Anti-mouse TCF7/TCF1-Alexa Fluor 888	S33-966	BD Bioscience
Anti-mouse TCF7/TCF1-Alexa Fluor 488	S33-966	BD Bioscience
Anti-mouse TCF7/TCF1-Alexa Fluor 888	CD63D9	Cell Signalling
Anti-mouse TIGIT-PE	1G9	Biolegend
Anti-mouse TIM-3-PE-Cy7	RMT3-23	Biolegend
Anti-mouse TIM3-BUV395	5D12	BD Bioscience
Anti-mouse TIM-3-BV785	RMT3-23	Biolegend
Anti-mouse TNF-α-PE	REA636	Miltenyi Biotech
Anti-mouse TOX-PE	E6G5O	Cell Signalling
Anti-mouse TOX Vio R667	REA473/TXRX10	Miltenyi Biotech

For intracellular staining, the “Intracellular Fixation & Permeabilization Buffer” kit was used according to the manufacturer’s instructions (Thermo Fisher Scientific) and for nuclear staining, the Foxp3/TF Staining Buffer Kit was utilized (Thermo Fisher Scientific). Finally, cells were resuspended in 100 µl of FACS buffer and acquired on the flow cytometer (BD FACs Canto II).

### RNA bulk sequencing

The RNA Seq was done in collaboration with Dr. Thorsten Bischler from the Core Unit Systems Medicine Würzburg. The RNA preparation was carried out using the RNeasy Plus Mini Kit (QIAGEN) according to the manufacturer’s protocol. The RNA quality was checked using a 2100 Bioanalyzer with the RNA 6000 Nano kit (Agilent Technologies). The RNA integrity number (RIN) for all samples was > 8.3. cDNA libraries were prepared from total RNA with TruSeq mRNA Stranded Library Prep Kit from Illumina according to manufacturer’s instructions (1/2 volume). Libraries were quantified by QubitTM Flex Fluorometer (ThermoFisher), and their quality was checked using the 2100 Bioanalyzer with DNA 1000 Kit (Agilent). The average size of the pool was approximately 300bp. Sequencing of pooled libraries, spiked with 1% PhiX control library, was performed at 25million reads/sample in single-end mode with 100nt read length on the NextSeq 2000 platform (Illumina). The data processing step was carried out with Illumina NextSeq RTA v3.9.25. FASTQ conversion with bcl2fastq v2.20.0.422. FASTQ quality and adapter trimming using Cutadapt (41) version 2.5 (cutadapt parameters:--nextseq-trim=20 -m 1 -a AGATCGGAAGAGCACACGTCTGAA CTCCAGTCAC). Alignment of trimmed reads to mouse genome (GRCm39) using STAR (42) v2.7.2b with default parameters based on RefSeq annotation version 109. Calculation of read counts on exon level summarized for each gene (RefSeq annotation version 109 for GRCm39) using feature Counts v1.6.4 from the Subread package (43). Multi-mapping and multi-overlapping reads were counted strand-specific and reversely stranded with a fractional count for each alignment and overlapping feature (command line parameters: -s 2 -t exon -M -O --fraction) and assembled with GRCm39. The statistical analysis and graphs were performed with R-studio.

### Immunoblots

For protein extraction, cells were harvested on d6 at a concentration of 4.5-5x106 cells per sample. They were washed with 1 ml of PBS and then centrifuged at maximum speed (14,000 rpm) for 5min using the Centrifuge 5418. Subsequently, the cells were carefully re-suspended in 100µl of IP buffer containing 0,1 M PMSF and incubated on ice for 30min. The samples were then placed on dry ice for 5min, thawed, and refrozen on dry ice for another 5min. Following this, 6 µl of 5 M NaCl was added to achieve a final concentration of 450mM, and the samples were vortexed and shaken for 30min in a cold room. Afterward, the samples were centrifuged again at maximum speed (14,000 rpm) for 30min. The supernatant was then transferred to fresh tubes. Nuclear and cytoplasmic extracts were prepared as described previously (44), antibodies for detection are given in **Table 2**.

**Table 2:** Primers for RT-PCR.

Antibody name	Clone	Company	Dilution
Goat anti-mouse IgG		Thermo Fisher	1:20000
Goat anti-rabbit IgG		Thermo Fisher	1:20000
Mouse anti-NFATc1	7A6	BD Pharmigen™	1:10000
Rabbit anti-NFATc1α	IG-457	ImmunoGlobe	1:1000
Rabbit anti-NFATc2		Sigma-Aldrich	1:10000
Goat anti-IRF-4	M-17	SCBT	1:10000

For protein quantification Bio-Rad Protein Assay Dye Reagent and a LUMIstar Omega microplate reader were used. Western blotting was performed as previously described (13, 14), detected by the “SuperSignal™ West Pico PLUS” kit according to the manufacturer’s protocol and were measured on the “Fusion SL” machine. As loading control, filters were stained by Ponceau Red.

### Real-time-PCR

The amplification of cDNAs in RT-PCR assays was quantified with SYBR Green as described before (45), primers are listed in **Table 3**.

**Table 3:** Antibodies for immunoblots.

Gene	Primers sequence
m*Actb*	fw 5’-TGTCCACCTTCCAGCAGATGT-3’ rev 5’-AGCTCAGTAACAGTCCGCCTAG-3’
m*Nfatc1* E1-E3 P1-driven transcripts	fw 5’-GGGAGCGGAGAAACTTTGC-3’ rev 5’-CAGGGTCGAGGTGACACTAGG-3’
m*Nfatc1* E2-E3 P2-driven transcripts	fw 5’-AGGACCCGGAGTTCGACTTC-3’ rev 5’-GCAGGGTCGAGGTGACACTAGG-3’
m*Nfatc1* E6-E7 all transcripts	fw 5’-GCCTCGAACCCTATCGAGTG-3’ rev 5’-AGTTATGGCCAGACAGCACC-3’
m*Nfatc2*	fw 5’-GGGTTCGGTGAGTGACAGTT-3’ rev 5’-CTCCTTGGCTGTTTTGGGATA-3’
m*Nfatc3*	fw 5`-AATCTTCCCTCAGTCCTAGTCC-3` rev 5`-GGTGAACCAAGAGTGAATCGTG-3`
m*Tcf7*	fw 5’-AGCTTTCTCCACTCTACGAACA-3’ rev 5’-AATCCAGAGAGATCGGGGGTC-3’
m*Pdcd1*	fw 5’-AAACCAGAAGGCCGGTTTCA-3’ rev 5’-GCTCCTCCTTCAGAGTGTCG-3’
m*Ifng*	fw 5’-TTGCCA AGTTTGAGGTCAACAA-3’ rev 5’-CGCTTCCTGAGGCTGGATTC-3’
*Havcr2*	fw 5’-TCAGGTCTTACCCTCAACTGTG-3’ rev 5’-GGGCAGATAGGCATTTTTACCA-3’
*Slamf6*	fw 5’-CAGCTAATGAATGGCGTTCTAGG-3’ rev 5’-CTTAGGTTGATAACGAGGGCAG-3’

## Results

### NFATc1/αA RNA induction in exhausted CD8^+^ T cells

**Figure 1:**
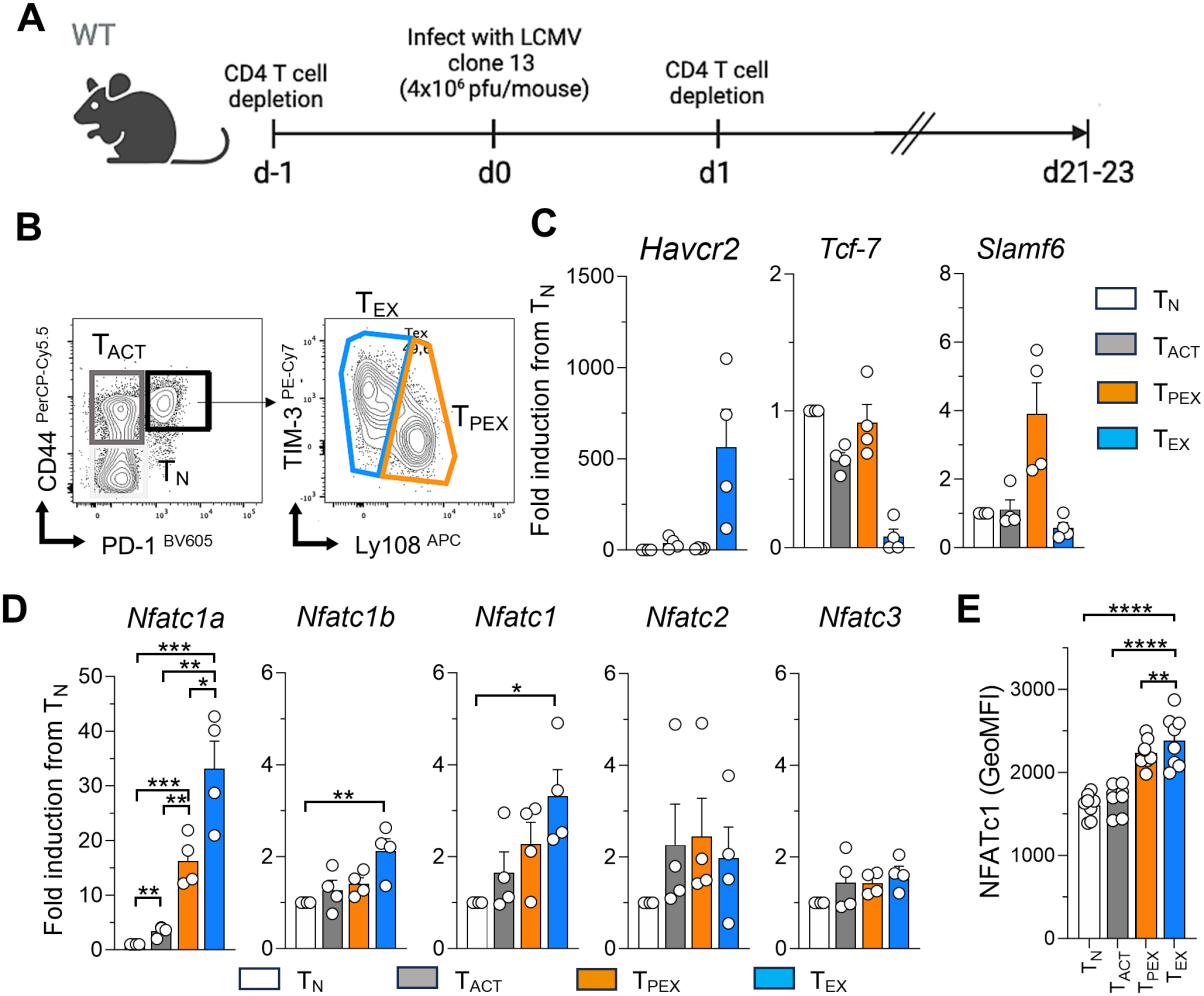
NFATc1 RNA induction in exhausted CD8^+^ T cells. **(A)** Scheme of chronic viral infection in mice with the lymphocytic choriomeningitis virus (LCMV) strain clone 13, in which CD4^+^ T cells were depleted one day before and one day after the infection to mount an isolated, endogenous CD8^+^ T-cell response. **(B)** Flow cytometric strategy to isolate naïve (CD44^–^), activated (CD44^+^PD-1^–^), T_PEX_ (CD44^+^PD-1^+^Ly108^+^TIM-3^–^) and T_EX_ (CD44^+^PD-1^+^Ly108^-^TIM-3^+^) CD8^+^ T cells from chronically infected mice by fluorescence-activated cell sorting (FACS). **(C)** Analysis of *Havcr2* (TIM-3), *Tcf7* (TCF-1) and *Slamf6* (Ly108) expression in FACS-sorted naïve, activated, T_PEX_ and T_EX_ cells using qRT-PCR; means +/- SEM of n=4 mice. Data were normalized to naïve T cells. **(D)** Analysis of P1 promoter-directed *Nfatc1a* and P2 promoter-directed *Nfatc1b* isoforms, as well as total *Nfatc1*, and *Nfatc2* and *Nfatc3* transcripts in FACS-sorted naïve, activated, T_PEX_ and T_EX_ cells using qRT-PCR; means +/- SEM of n=4 mice. Statistical analysis was performed using unpaired two-tailed Student’s *t*-test: *p<0.05; **p<0.01; ***p<0.001. **(E)** Intracellular staining of NFATc1 (by anti-NFATc1 7A6) and flow cytometric analysis in the sorted cells (C+D). A paired Student’s *t*-test was performed: **p<0.01; ***p<0.001; ****p<0.0001.

In a previous publication using acute *versus* chronic LCMV infection, a marked increase in NFATc1/αA and decrease in NFATc2 proteins was detected in exhausted CD8^+^ T cells (22). To examine whether these changes in NFAT proteins were generated at the transcriptional level, we used specific primers for the detection of individual *Nfat* RNAs in CD8^+^ T cells isolated from chronically LCMV-infected mice. In qPCR assays, various NFAT transcripts were determined in flow cytometry-sorted T_PEX_ and T_EX_ cells and compared to those in naïve as well as in activated CD8^+^ T cells (**Figures 1A, B**). The stage of the sorted cells was verified by their *Havcr2*, *Tcf7*, and *Slamf6* RNA expression (**Figure 1C**). We detected a 10-20-fold increase in *Nfatc1a* RNA levels, encoding the short isoform NFATc1/αA, in T_PEX_ and in T_EX_ cells, compared to resting T cells. In striking contrast, no – or very subtle – changes in *Nfatc2* and *Nfatc3* RNA levels were observed (**Figure 1D**). Reanalysis of our single-cell RNA sequencing data from CD8^+^CD44^+^PD-1^hi^ T cells in chronically LCMV-infected mice confirmed the dominant expression of *Nfatc1* RNA in exhausting cells and its pronounced upregulation in T_EX_ cells (**Supplementary Figures 1A, B**). *Nfatc2* was only marginally detectable in T_PEX_ cells, while *Nfatc3* persisted and seemed to characterize CTL-like CD8^+^CD44^+^PD-1^hi^ T cells. *Nfatc4* could not be observed in exhausting CD8^+^ T cells, consistent with the concept that NFATc4 is rarely expressed in lymphocytes (46). Even though it is not activated by TCR/Ca²^+^/CN signaling, we also monitored *Nfat5*, playing a negative role in T-cell exhaustion (47) (**Supplementary Figure 1C**). Importantly, an overall increase of NFATc1 protein in T_PEX_ and even more in T_EX_ cells could be verified by intracellular staining and flow cytometry (**Figure 1E**). These findings indicate that in exhausted CD8^+^ T cells NFATc1 is strongly induced, whereas *Nfatc2* and *Nfatc3* transcription remained at basal or unchanged levels. Further, as determined by *Nfatc1a* RNA, the isoform NFATc1/αA was predominant in T_EX_ cells.

### NFATc1 and NFATc2 control the survival and exhaustion of virus-specific T cells

**Figure 2:**
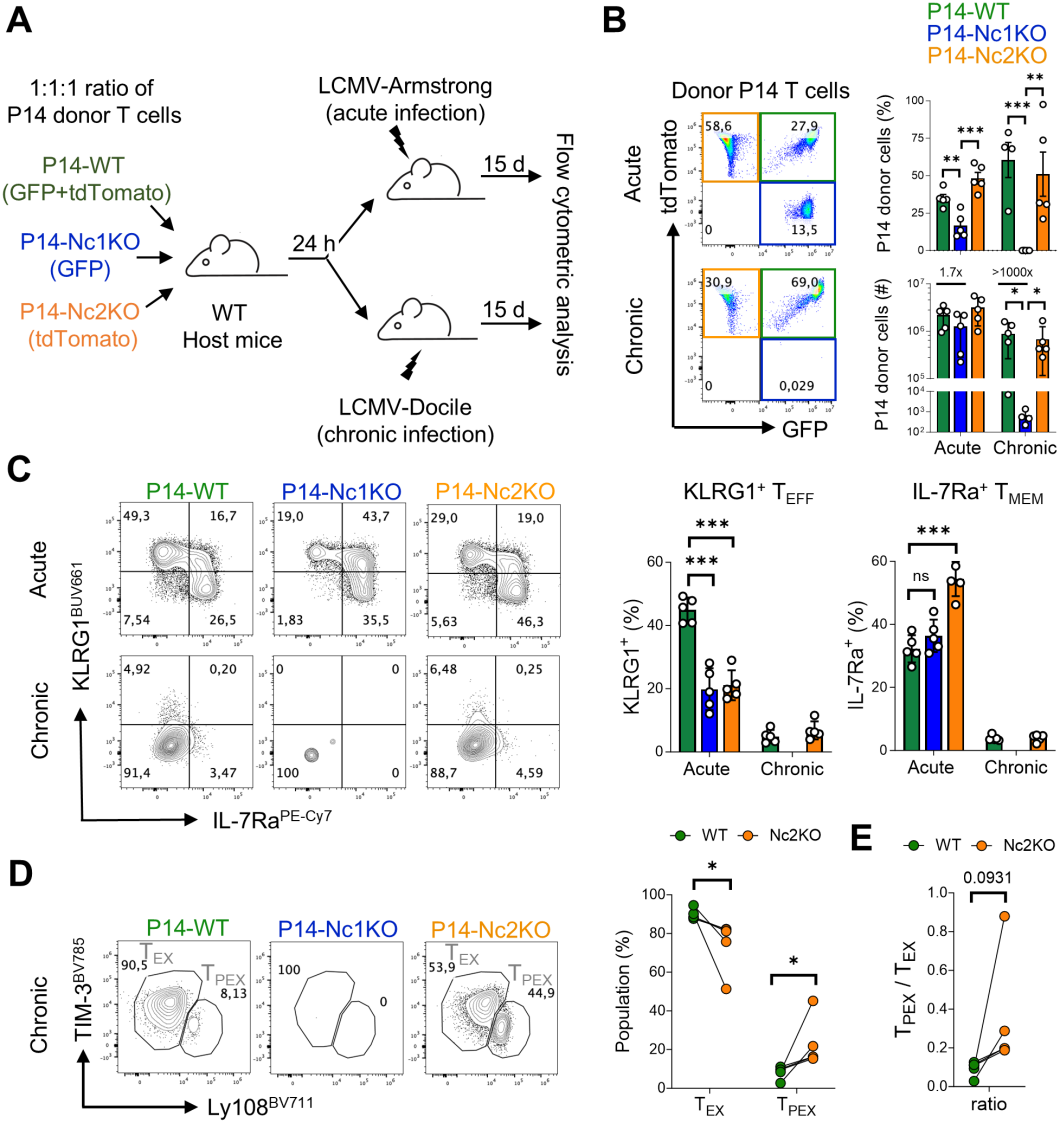
NFATc1 and NFATc2 regulate the survival and differentiation of virus-specific CD8^+^ T cells upon chronic viral infection. **(A)** Scheme of the adoptive co-transfer experiments. Equal numbers of WT (GFP^+^/tdTomato^+^), NFATc1-deficient (GFP^+^), and NFATc2-deficient (tdTomato^+^) P14 donor CD8^+^ T cells were co-transferred into WT recipient mice, followed by (acute) LCMV-Armstrong or (chronic) LCMV-Docile infection. **(B)** Frequency and absolute number of donor P14 T cells in the spleen at 15 days post infection (d.p.i.) with LCMV-Armstrong or LCMV-Docile. Means ± SEM from 5 mice. **(C)** Flow cytometric analysis of KLRG1^+^IL-7Rα^-^ effector and KLRG1^-^IL-7Rα^+^ memory precursor T cell subsets among donor P14 cells following acute and chronic LCMV infection; means ± SEM of 5 mice. **(D)** Quantification of Ly108^+^TIM-3^-^ T_PEX_ and Ly108^-^TIM-3^+^ T_EX_ P14 T cells in chronically LCMV-Docile infected mice by flow cytometry; means ± SEM of 5 mice. **(E)** Ratio of T_PEX_ to T_EX_ cells in P14 donor populations as quantified in **(D)**. Statistical analysis in **(B–E)** was performed using unpaired two-tailed Student’s *t*-test: *p<0.05; **p<0.01; ***p<0.001.

To dissect the roles of NFATc1 and NFATc2 in virus-specific CD8^+^ T-cell responses during acute and persistent LCMV infection, we generated NFATc1- and NFATc2-deficient mice on a P14 TCR-transgenic background (48). These animals express an antigen receptor specific for the LCMV GP_33–41_ epitope and distinct fluorescent reporters. To evaluate cell-intrinsic functions of NFATc1 and NFATc2, we performed adoptive transfer experiments in which equal numbers of WT (GFP^+^/tdTomato^+^), NFATc1-deficient (GFP^+^) and NFATc2-deficient (tdTomato^+^) P14 CD8^+^ T cells were co-transferred into WT-recipient mice (49). The host mice were subsequently infected with either LCMV-Armstrong (for acute infection) or LCMV-Docile (chronic infection), and donor T-cell expansion and differentiation were monitored over the course of 15 days post infection (d.p.i.) (**Figure 2A**). Following acute LCMV infection, NFATc1-deficient T cells exhibited a moderate reduction in the spleen compared to WT controls. In contrast, during chronic LCMV infection, NFATc1-deficient P14 cells were nearly undetectable by 15 d.p.i., indicating a critical role for NFATc1 in T-cell survival under persistent antigen stimulation (**Figure 2B**). Both NFATc1 and NFATc2 were required for the differentiation of short-lived KLRG1^+^IL-7Rα^–^ effector T cells during acute infection. However, NFATc2 deficiency resulted in a marked enhancement of KLRG1^–^IL-7Rα^+^ memory precursor T-cell differentiation, suggesting that NFATc2 controls effector *versus* memory fate decisions (**Figure 2C**).

In the context of a persistent LCMV-Docile infection, canonical effector and memory differentiation were not observed (**Figure 2C**). Instead, persistent antigen exposure drives the development of T_EX_ cells (17). These are characterized by reduced proliferation, diminished cytokine production and impaired cytolytic function. However, a T_PEX_ progenitor population sustains the terminal T_EX_ cell pool (20). T_PEX_ cells can be identified by different memory-associated markers such as Ly108 (gene name *Slamf6*), and the absence of terminal exhaustion markers like TIM-3 (*Havcr2*) (49). Strikingly, NFATc2-deficient P14 T cells displayed an increased frequency of Ly108^+^TIM-3^-^ T_PEX_ cells and a corresponding reduction in Ly108^-^TIM-3^+^ terminally T_EX_ cells following LCMV-Docile infection (**Figures 2D, E**). These findings demonstrate that NFATc1 is essential for the survival of virus-specific T cells during chronic viral infection, whereas NFATc2 promotes their progression toward terminal exhaustion.

### NFATs control the exhaustion of CD8^+^ T cells *in vitro*

The poor survival of NFATc1-deficient T cells in exhausted T cells *in vivo* prompted us to generate exhausted CD8^+^ T cells *in vitro* for mechanistic investigations. To this end, we followed an *in vitro* protocol that was published previously (50). Isolated CD8^+^ T cells were stimulated with plate-bound anti-CD3/CD28 Abs (plus IL-2) for two days and then cultured for six days under “exhausting” or “acute” conditions. Under exhausting conditions, the cells were persistently stimulated with anti-CD3 Abs every other day, while under acute conditions, they remained unstimulated after day 2, to be cultured in IL-2-containing medium only (**Supplementary Figure 2A**). The two types of CD8^+^ T cells differed markedly in their proliferation and cell death rate over six days. Whereas acute stimulation induced logarithmic expansion of CD8^+^ T cells with fewer than 5% dying cells, chronically activated CD8^+^ T cells ceased to proliferate. Instead, more than 30% of cells had died within 6 days of culture under chronic conditions, and the majority were dead after 8–10 days of incubation (**Supplementary Figures 2B–D**).

Importantly, the two types of CD8^+^ T cells differed markedly in the expression of various exhaustion markers. While in more than 90% of chronically stimulated CD8^+^ T cells PD-1 and LAG-3 were highly expressed, much less cells expressed those exhaustion marker proteins upon acute stimulation, and a similar dichotomy between chronic and acute stimulation was detected for TIM-3 (**Supplementary Figure 2E**). When these cells of acute stimulation were stimulated by TPA and ionomycin (T+I) for one hour, a strong 144-fold induction of *Ifng* RNA was observed, whereas due to the high basal level upon chronic stimulation only a 2-3-fold increase in *Ifng* induction was found. This resulted in a much higher *Pdcd1* expression, but less *Ifng* and *Il2* expression, in T+I restimulated cells when kept under chronic *versus* acute conditions before (**Supplementary Figures 2F, G**).

To determine the role of NFAT factors in generating *in vitro* exhausted cells, we investigated the gene expression profiles of WT and NFATc1c2-double-deficient T cells stimulated under chronic conditions. Although the general activation potential to drive T-cell memory was preserved on day 2 (**Supplementary Figure 3A**), inactivation of both the *Nfatc1 and Nfatc2* genes resulted in a strong decrease in the RNA levels of the exhaustion marker genes *Pdcd1, Lag3*, and *Tigit* in chronically stimulated CD8^+^ T cells, demonstrating an important role of NFATs in the generation of CD8^+^ T-cell exhaustion (**Supplementary Figure 3B**). Still, similar RNA expression of CD44, CD69, and the proliferation marker Top2a, as well as protein expression of CD44 and CD69, between WT and DKO cells ensured the overall responsiveness to stimulation of NFATc1 and NFATc2 double-deficient CD8^+^ T cells (**Supplementary Figures 3C, D**).

### Impaired survival of NFATc1-deficient CD8^+^ T cells under chronic antigenic stimulation

**Figure 3:**
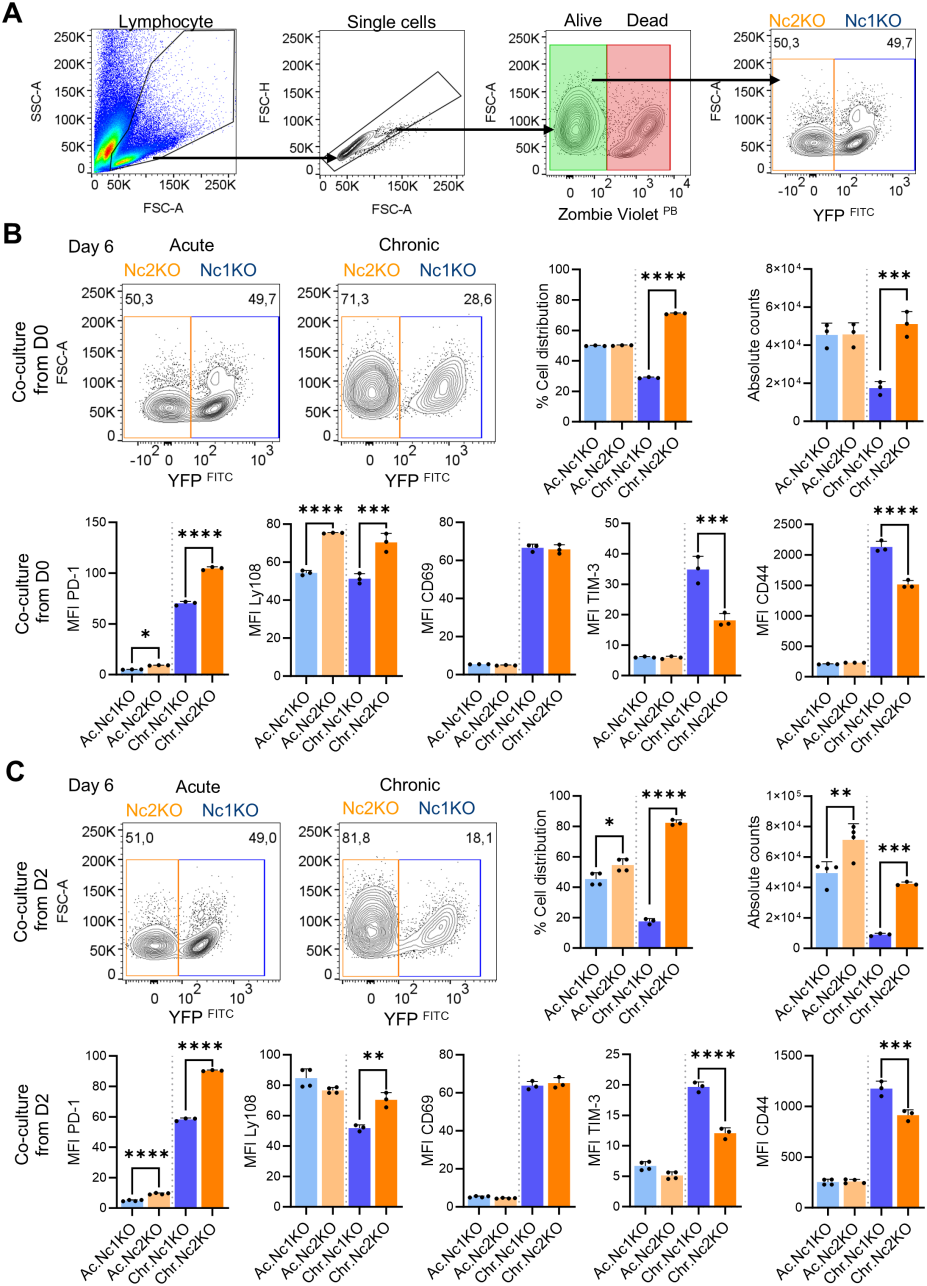
NFATc1 supports the survival of chronically stimulated CD8^+^ T cells *in vitro.* **(A)** Scheme of strategy of flow cytometry. **(B)** Co-incubation assays. 5x10^5^ YFP NFATc1- and NFATc2-deficient T cells were mixed, co-stimulated for 2 days and co-incubated for 4 days under acute and chronic conditions (see **Supplementary Figure 1A**). At d6, the composition of cultures was determined by flow cytometry. The column presentations show the absolute distribution and numbers of cells from 3 assays after co-incubation. In addition, they show the expression levels of PD-1, Ly108, CD69, TIM-3 and CD44. Data from 3 co-incubations are shown. **(C)** YFP NFATc1- and NFATc2-deficient cells were stimulated for 2 days in separated wells followed by co-incubation for 4 days [and flow cytometry as in **(B)**]. Statistical analysis in (B+C) was performed using unpaired two-tailed Student’s *t*-test with *Welch*’s correction: *p ≤ 0.05; **p<0.01; ***p<0.001; ****p<0.0001.

The disappearance of NFATc1-deficient CD8^+^ T cells upon adoptive transfer into mice challenged by chronic LCMV urged us to follow the survival of WT, NFATc1- and NFATc2-deficient CD8^+^ T cells in co-culture assays. When we mixed isolated NFATc1-, and NFATc2-deficient CD8^+^ T cells at the start of assays in a ratio 1:1 and incubated the cells under chronic conditions, we detected a strong decrease in NFATc1-deficient cells to approximately one half of NFATc2-deficient cells after six days. By contrast, no – or a very moderate – loss of NFATc1-deficient cells was observed in co-incubations under acute stimulation conditions (**Figures 3A, B**). Similar results were obtained when NFATc1- and NFATc2-deficient cells were mixed after two days of initial stimulation followed by co-incubation for four days (**Figure 3C**), or when NFATc1-deficient cells were mixed and co-incubated with WT CD8^+^ T cells (**Supplementary Figure 4**). Regardless of whether the cells were co-cultivated from the beginning or only after the initial stimulation, the NFATc1- and NFATc2-deficient cells differed in the expression of PD-1, Ly108, TIM-3 and CD44, whereas for the activation marker CD69, a strong but very similar increase was detected in CD8^+^ T cells upon chronic activation (**Figures 3B, C**).

Collectively, these findings demonstrate that NFATc1 induction contributes to the survival of CD8^+^ T cells under persistent antigenic stimulation *in vitro.*

### NFATc2 supports T_EX_ marker genes

**Figure 4:**
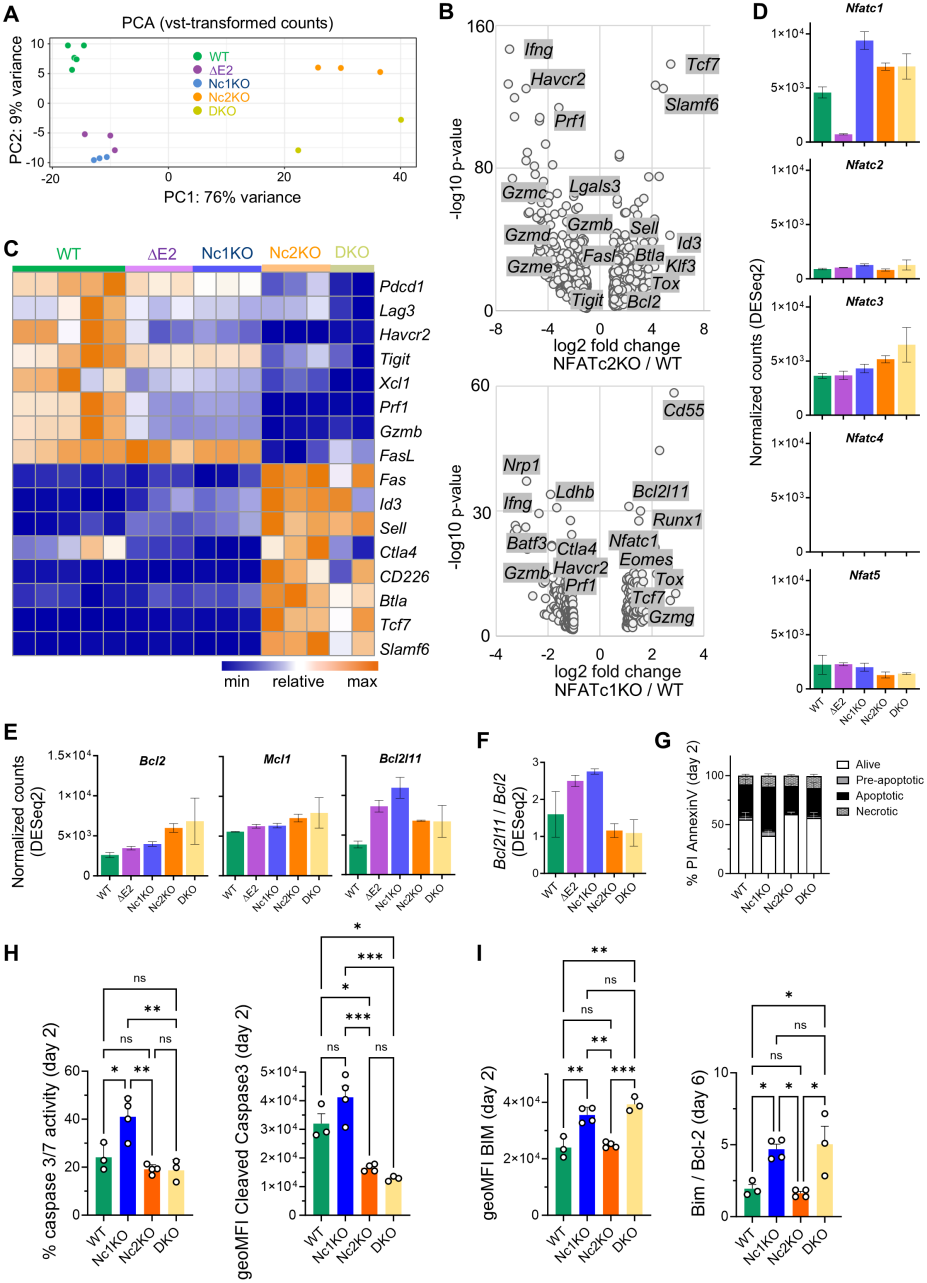
Transcriptomic profiling of NFAT-deficient CD8^+^ T cells under chronic *in vitro* stimulation. **(A)** Principal component analysis (PCA) of results of bulk NGS assays with exhausted CD8^+^ T cells from WT, ΔE2-, NFATc1-, NFATc2-, and NFATc1+NFATc2-double-deficient mice generated *in vitro*. **(B)** Volcano plots of results of bulk NGS assays of CD8^+^ T cells from either NFATc2- (above) or NFATc1-deficient CD8^+^ T cells (below) compared to WT. **(C)** Module of prominent genes that were either suppressed (in blue) or enhanced (orange) in expression upon inactivation of E2 enhancer (ΔE2), of NFATc1, NFATc2, or of both NFAT genes (DKO) in CD8^+^ T cells. **(D)** Transcriptomic expressions of *Nfatc1*, *Nfatc2*, *Nfatc3*, *Nfatc4*, and *Nfat5*. **(E)** Individual depiction of the RNA expression of *Bcl2*, *Mcl1*, and *Bcl2l11*. **(F)** Calculated RNA ratios of *Bcl2l11*/*Bcl2*. **(G, H)** Flow cytometric analyses of WT, NFATc1KO, NFATc2KO, and DKO CD8^+^ T cells after two days of anti-CD3/CD28 + IL-2 stimulation to determine the distribution of live, preapoptotic, apoptotic, and necrotic cells **(G)** and the frequency of caspase 3/7^+^ cells and cleaved caspase 3 expression per cell **(H, I)** Flow cytometric evaluation of Bim expression on day 2 and the Bim/Bcl2 ratio on day 6 in chronically stimulated WT, NFATc1KO, NFATc2KO, and DKO CD8^+^ T cells.

To elucidate the role of NFAT factors in gene regulation during chronic stimulation, we performed bulk RNA-sequencing (RNA-seq) to determine the transcriptomes of WT, NFATc1-, NFATc2-, and NFATc1c2-double deficient T cells under chronic stimulation. In addition, we included in these assays CD8^+^ T cells from a mouse line designated as ΔE2, which bears a “floxed” E2 enhancer (38). Upon crossing with *Cd4-cre* mice, in both CD4^+^ and CD8^+^ T cells from those mice, the E2 enhancer was efficiently deleted, and the expression of all NFATc1/α isoforms, particularly NFATc1/αA, selectively abolished (**Supplementary Figures 5A–C**). The overall gene expression signatures – documented by principal component analysis (PCA) and heat map presentation (**Figure 4A**; **Supplementary Figure 6A**) – revealed only moderate differences between WT, ΔE2, and NFATc1-deficient T cells. In stark contrast, numerous genes were significantly altered between NFATc2-deficient or NFATc1c2 double-deficient T cells compared to WT and NFATc1-deficient lymphocytes (**Figure 4A**). These findings suggest that NFATc1 and NFATc2 exert distinct effects on *in vitro* exhausted CD8^+^ T cells.

A more detailed view on the molecular changes between NFATc1- and NFATc2-deficient T cells indicated that NFATc2 suppresses the expression of numerous genes that are characteristic for progenitor CD8^+^ T_PEX_ cells, including the *Fas, Id3, Sell, Cd226, Btla, Tcf7*, *Slamf6*, and *Klf2* (51), as well as *Dapl1*, whose gene product negatively regulates NFATc2 activity (52) (**Figure 4B**; **Supplementary Figure 6B**). By contrast, the expression of genes that are typically upregulated in terminally differentiated T_EX_ cells, such as the *Pdcd1, Tigit, Havcr2, Gzmb, Xcl1*, *Prf1*, *Nr4a2*, *Zeb2*, and *Rbpj*, was diminished in NFATc2-deficient CD8^+^ T cells under chronic stimulation (**Figures 4B, C**; **Supplementary Figure 6C**). Of note, the differential gene expression in the absence of NFATc2 was reflected in a shifted distribution of T_PEX_ to T_EX_ cells after 6 days of chronic stimulation (**Supplementary Figure 6D**). In the absence of NFATc1, we only observed a tendency towards an upregulation of T_PEX_ genes and decrease in T_EX_ transcripts. Most T_EX_ marker genes remained largely unchanged in NFATc1-deficient and ΔE2 cells. However, expression of others, including *Pdcd1*, *Gzmb*, and *Prf1*, showed moderate decreases. Interestingly, NFATc1 deficiency was counterregulated by an increase in *Nfatc1* RNA, whereas the deletion of the enhancer E2 – responsible for all inducible, exon1-encoded, isoforms – abolished most *Nfatc1* RNA otherwise expressed upon chronic stimulation (**Figure 4D**).

According to cell trace dilution and the frequency of Ki67^+^ cells, chronically activated NFATc1-deficient CD8^+^ T cells were hardly able to proliferate, whereas NFATc2-deficient cells had an advantage (**Supplementary Figures 6E, F**). Among the genes that were slightly increased in NFATc1-deficient CD8^+^ T cells was the *Bcl2l11* gene, encoding the pro-apoptotic BH3-only protein, Bim (53). Considering the elevated ratio of *Bcl2l11* to *Bcl2*, the later encoding the survival protein Bcl-2 (**Figures 4E, F**), it seems not unlikely that such differences would contribute to the rapid loss of NFATc1-deficient cells during the exhaustion of CD8^+^ T cells. Accordingly, already as early as on day 2, the frequency of apoptotic cells, caspase 3/7^+^ cells, and the mean fluorescence intensity (MFI) of caspase 3 were highest in NFATc1-deficient CD8^+^ T cells compared to WT, NFATc2KO, or DKO cells (**Figures 4G, H**). Similarly, Bim protein was enriched in NFATc1-deficient CD8^+^ T cells early on, resulting in an increased Bim-to-Bcl-2 ratio by day 6 of chronic stimulation (**Figure 4I**).

Overall, unbiased transcriptomic analyses, supported by protein data acquired by flow cytometry, revealed that NFATc1 and NFATc2 play distinct – and, in some cases, opposing – roles in regulating gene expression during chronic antigenic stimulation.

### NFATc2 drives CD8^+^ T-cell exhaustion

**Figure 5:**
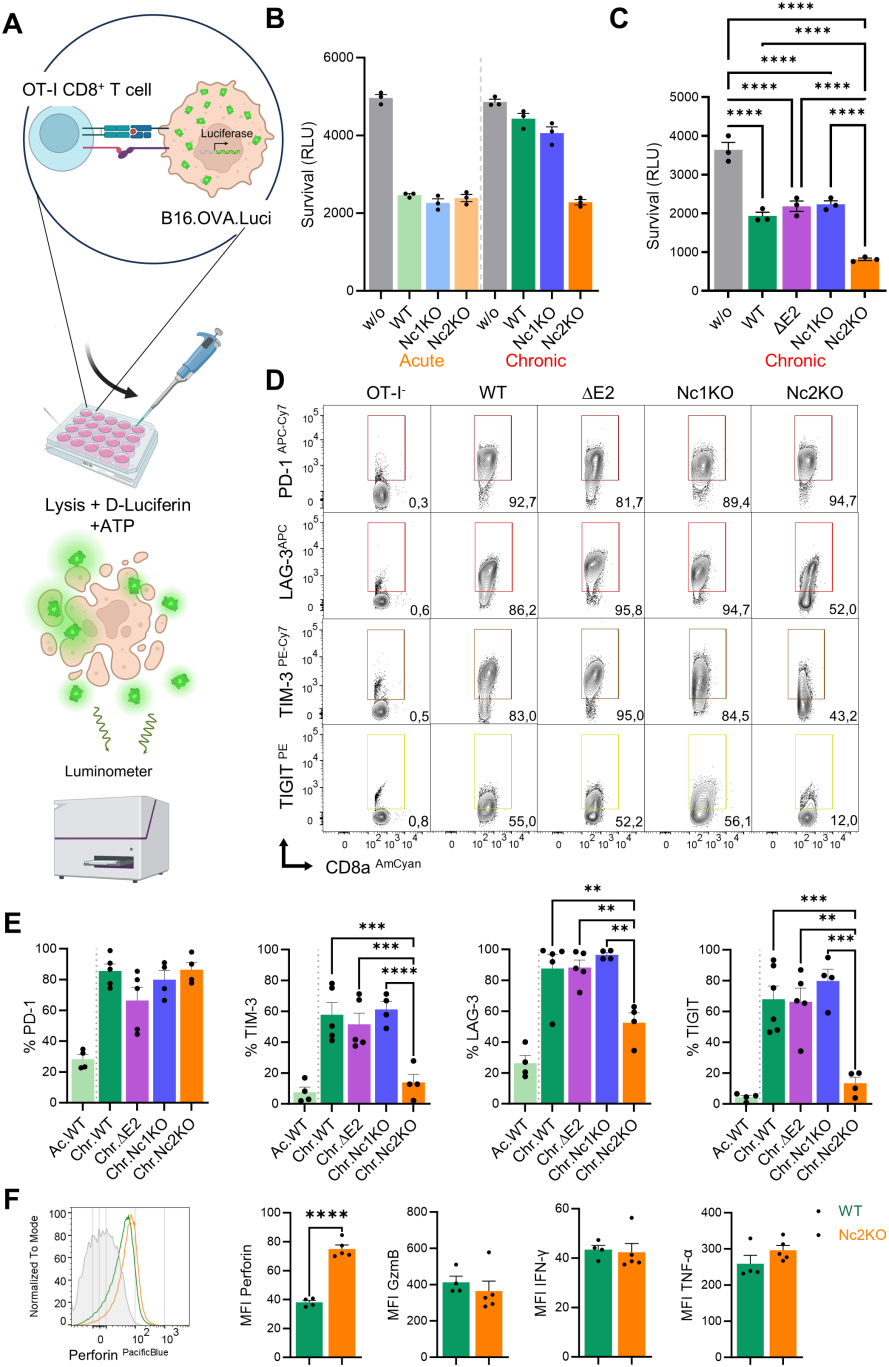
NFATc2 promotes terminal exhaustion of CD8^+^ T cells *in vitro*. **(A)** Scheme of the killing of B16 OVA Luci tumor cells by OT-I CD8^+^ T cells *in vitro*. **(B, C)** Quantitation of killing activity of WT and various NFAT-deficient CD8^+^ T cells in co-incubations with tumor cells for 18h, measured in triplicates, **(C)** n=3. The activity of acutely or chronically activated CD8^+^ T cells is shown. **(D, E)** Expression of the exhaustion markers PD-1, TIM-3, LAG-3 and TIGIT on OT-I CD8^+^ T cells upon co-incubation with tumor cells as shown in representative dot plots **(D)** and quantitated in column diagrams **(E, F)** Representative graph of normalized MFI of perforin and summary plots for Prf-1, GzmB, IFN-γ, and TNF-α expression in chronically activated WT *versus Nfatc2*^–/–^ OT-I CD8^+^ T cells upon co-incubation with tumor cells. Statistical analysis was performed using unpaired two-tailed Student’s *t*-test with *Welch*’s correction: **p<0.01; ***p<0.001; ****p<0.0001.

To determine the individual roles of NFATc1 and NFATc2 in T-cell exhaustion, we conducted co-incubation assays using B16 melanoma cells that express hen egg ovalbumin (OVA) and luciferase with TCR transgenic OVA-specific OT-I T cells (**Figure 5A**). In those assays, chronically stimulated OT-I T cells showed a poor killing activity, compared to acutely stimulated T cells. Acutely stimulated CD8^+^ T cells, deficient in either NFATc1 or NFATc2, were as potent in tumor cell killing as their WT counterparts. By contrast, chronically stimulated NFATc1-deficient CD8^+^ T cells killed tumor cells almost as poorly as WT T_EX_ cells, whereas “exhausted” NFATc2-deficient T_EX_ cells eradicated tumor cells as efficiently as acutely stimulated CD8^+^ T cells (**Figure 5B**).

We also included OT-I transgenic CD8^+^ T cells with ablation of the E2 enhancer (OT-I ΔE2 T cells) in our killing assays. Compared to chronically stimulated WT, NFATc1-, and NFATc2-deficient cells, the ΔE2 CD8^+^ T cells showed the same killing activity as WT and NFATc1-deficient T_EX_ cells, and expressed the checkpoint molecules PD-1, TIM-3, LAG-3 and TIGIT at the same frequency as WT and NFATc1-deficient cells. In marked contrast, the frequency of TIM-3^+^, LAG-3^+^ or TIGIT^+^ T_EX_ cells were lower among chronically stimulated NFATc2-deficient CD8^+^ T cells (**Figures 5C–E**). This correlated well with their enhanced killing capacity, particularly given that Prf-1 was expressed at significantly higher levels than in WT cells upon contact with tumor cells (**Figure 5F**). This suggested a higher potential of chronically stimulated NFATc2-deficient CD8^+^ T_PEX_ cells to be reinvigorated.

Similar results for NFATc2 in exhaustion were obtained when the OT-I CD8^+^ T cells were chronically stimulated with the OVA-peptide SIINFEKL instead of plate-bound anti-CD3/CD28 Abs. At the protein level, those NFATc2-deficient CD8^+^ T cells showed a marked increase in T-bet and a moderate increase in TCF-1 and PD-1, but a decrease in Eomes expression (**Supplementary Figures 7A, B**). These results suggest that NFATc2 drives terminal T-cell differentiation, as for example T-bet and Eomes are expressed during the acute and late/memory phases of CD8^+^ T-cell differentiation (54).

Another way to assess the level of exhaustion is the evaluation of TOX (24–26). In line, we verified higher levels of Tcf-1 in anti-CD3-treated NFATc2-deficient CD8^+^ T cells but showed reduced levels of TOX expression in chronically stimulated NFATc2KO and DKO (**Supplementary Figure 7C**).

Taken together, these data suggest that NFATc2 drives the differentiation of terminally exhausted CD8^+^ T cells during chronic stimulation *in vitro*, whereas NFATc1 – including its predominantly induced isoform, NFATc1/αA – supports the survival of these cells without a major contribution to the progression of exhaustion.

### NFATc1/αA induction interferes with NFATc2 expression in exhausted CD8^+^ T cells

**Figure 6:**
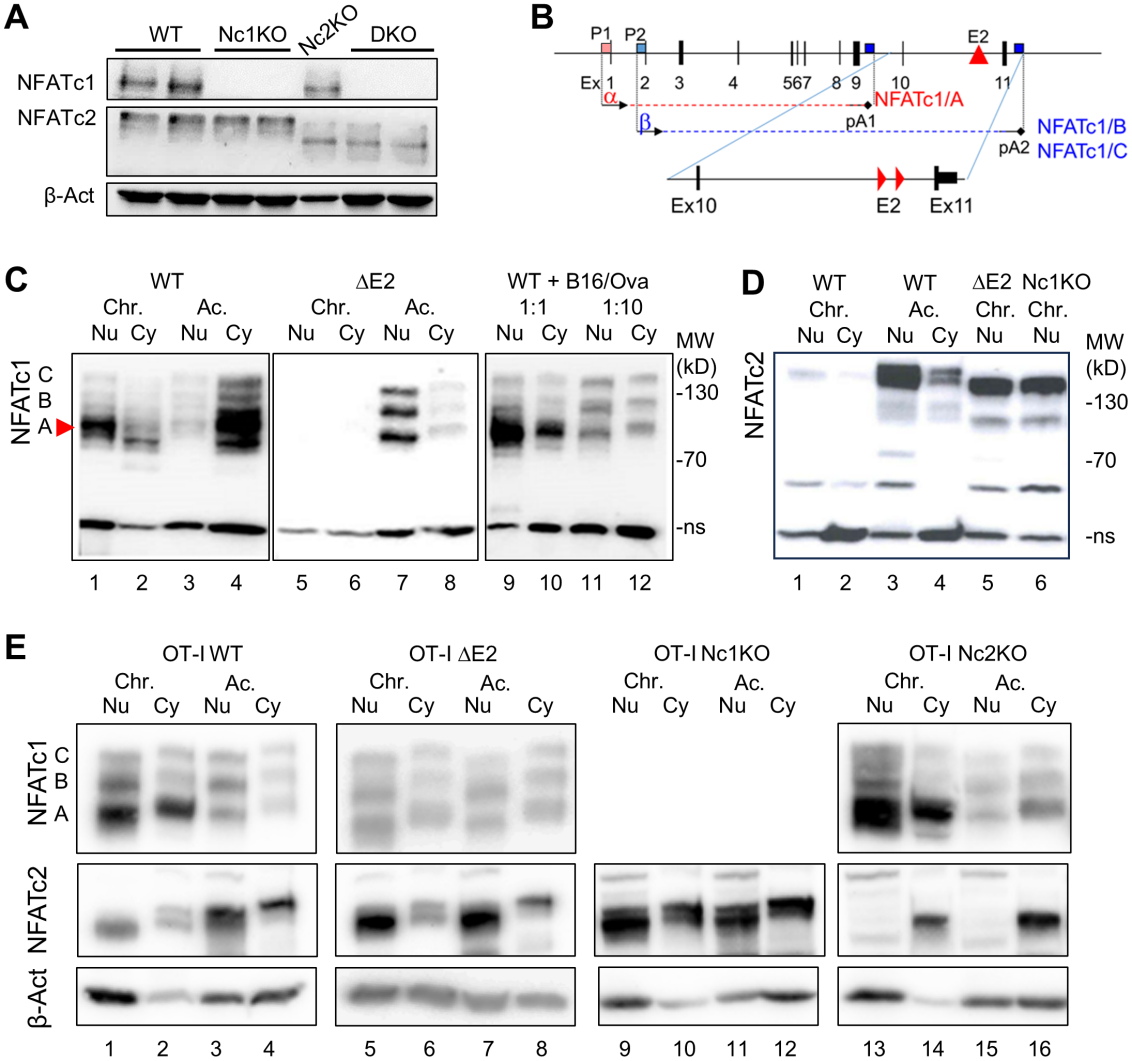
NFATc1/αA is predominantly expressed in chronically stimulated CD8^+^ T cells. **(A)** Immunoblots with whole cell extracts exposing the loss of all NFATc1 isoforms (due to 1^st^ common exon, i.e., exon3 deletion) and exon6-deleted NFATc2 in *Nfatc1*^–/–^ and/or *Nfatc2*^–/–^ CD8^+^ T cells, stimulated for two days. **(B)** The murine *Nfatc1* gene. The position of the two promoters, P1 (soft red) and P2 (soft blue), and of two polyA addition sites, pA1 and pA2 (dark blue boxes) are shown. The predominant isoforms are depicted: NFATc1/A (red dashed line) expression is directed by P1 and the enhancer E2 (red triangle) and includes the exon1-encoded α-peptide. The long isoforms NFATc1/B and NFATc1/C (blue dashed line) are driven by the constitutive promoter P2 which leads to exon2-encoded β-peptide expression. **(C)** Immunoblots for the detection of NFATc1 in nuclear (Nu) and cytosolic fractions (Cy) of WT or ΔE2 CD8^+^ T cells upon chronic (Chr.) or acute (Ac.) activation for 6 days (lanes 1-8). In lanes 9-12, OT-I T cells were co-incubated with B16/Ova melanoma cells for 5 days (in a ratio of T:tumor cells of 1:1 or 1:10). The NFATc1/αA band is highlighted by red arrows. Immunodetection with the 7A6 NFATc1 Ab that recognizes all NFATc1 proteins. **(D)** Immunoblots for the detection of NFATc2 in nuclear or cytosolic fractions from WT, ΔE2 or NFATc1-deficient CD8^+^ T cells upon chronic or acute activation. Immunodetection with an Ab directed against all NFATc2 proteins. **(E)** Immunoblots of nuclear (Nu) and cytosolic (Cy) protein extracts from OT-I CD8^+^ T cells stimulated with SIINFEKL peptide under acute (Ac.) and chronic (Chr.) conditions. Cells were derived from WT (lanes 1-4), ΔE2 (lanes 5-8), NFATc1- (lanes 9-12) or NFATc2 (lanes 12-16)-deficient mice and detected for NFATc1, NFATc2, and β-actin.

We verified the deletion of NFATc1 and NFATc2 in two-day-activated CD8^+^ T cells by immunoblotting (**Figure 6A**). The induction of NFATc1/αA is orchestrated by the inducible *Nfatc1* promoter P1, the weak proximal polyA site pA1, alternate splicing, and a remote enhancer, designated as E2, located within the last intron of the gene (45, 55) (**Figure 6B**; **Supplementary Figures 5A–C**). When we examined the expression of nuclear and cytosolic NFAT proteins from CD8^+^ T cells upon chronic or acute stimulation *in vitro* for six days, we observed the expression of nuclear NFATc1/αA upon chronic, but hardly by acute stimulation including four days of cultures with IL-2 only (**Figure 6C**, lanes 1-4). This was confirmed by the complete loss of the inducible NFATc1 proteins in CD8^+^ T cells from ΔE2 mice, in which, due to the deletion of E2 enhancer, NFATc1 induction was abolished, whereas other NFATc1 proteins directed by the constitutive P2 promoter were still synthesized upon acute stimulation (**Figure 6C**, lanes 5-8). A similar induction of NFATc1/αA was observed in OT-I CD8^+^ T cells upon co-culture with B16/OVA melanoma cells for five days (**Figure 6C**, lanes 9-12). Intriguingly, parallel to the strong induction of NFATc1/αA, we observed a repression of NFATc2 at the protein level in chronically stimulated cells compared to acute stimulation (**Figure 6D**, lanes 1 and 3). The reduction in NFATc2 expression in chronically stimulated cells was dependent on the presence of NFATc1/αA, as in ΔE2 and NFATc1-deficient cells – in which the induction of NFATc1/αA was abolished – we detected unaltered nuclear NFATc2 levels (**Figure 6D**, lanes 3, 5 and 6). Upon comparison of OT-I NFAT single-deficient T cells, we could document that not only chronically stimulated *Nfatc1*^–/–^ CD8^+^ T cells do not repress NFATc2 in T_EX_ cells, but that NFATc1 expression is enforced in *Nfatc2*^–/–^ CD8^+^ T cells (**Figure 6E**).

### NFATc2 ablation improves the anti-tumor activity of CD8^+^ T cells

**Figure 7:**
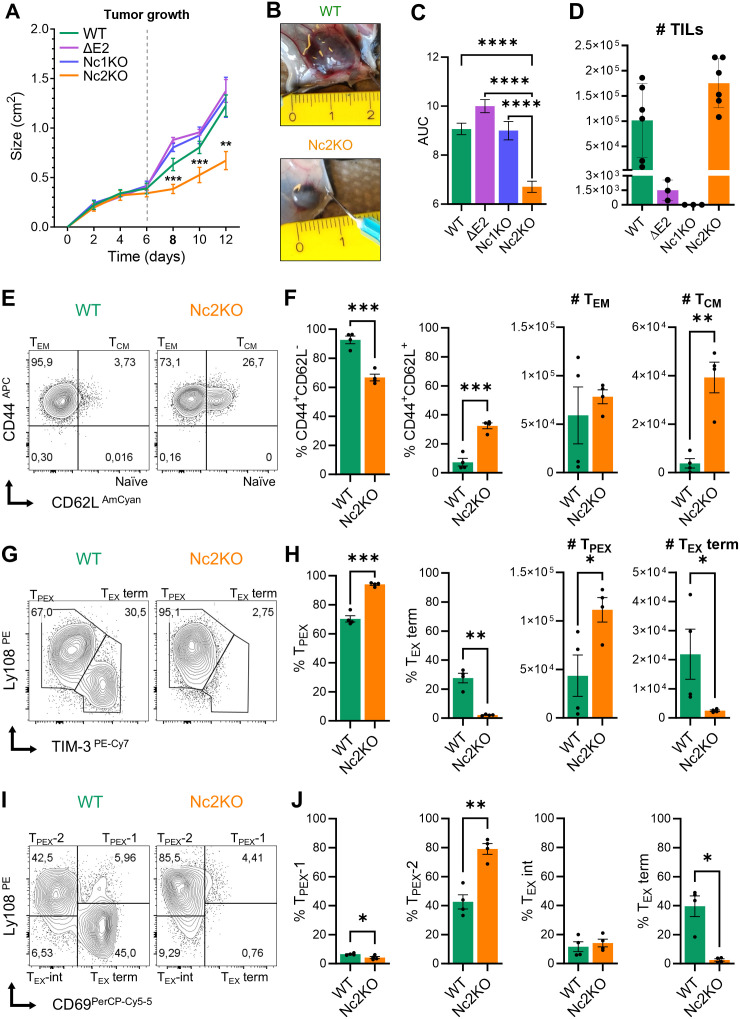
Loss of NFATc2 improves the antitumor activity of CD8^+^ T cells. **(A)** Time course of tumor growth of B16 melanomas upon injection at d6 (dotted vertical line) of WT (green line), ΔE2 (violet), NFATc1-deficient (blue) and NFATc2-deficient (orange) CD8^+^ T cells (orange) into *Rag1^–/–^* mice; negative control (no T-cell transfer) in grey; mean ± SEM. Data were collected from 3 control mice, 7 WT, 7 ΔE2, 7 NFATc1-deficient and 6 NFATc2-deficient mice. **(B)** Light microscopy of B16/Ova melanoma formation in *Rag1*^–/–^ mice with transferred WT or NFATc2-deficient CD8^+^ T cells 12 days after tumor inoculation. **(C)** Time course of tumor growth area under the curve (AUC) of the tumor sizes at d12 [as shown in **(A)**]. **(D)** Number of WT, ΔE2, NFATc1- and NFATc2-deficient cells in the population of TILs. (E+F) Characterization of TILs by flow cytometry using the T_EM_ and T_CM_ (central memory) markers CD44 and CD62L, frequencies and absolute numbers, respectively. (G+H) Characterization of TILs by the Ly108 and TIM-3 markers in flow cytometry assays. (I+J) Characterization of Ly108^+^CD69^+^ T_PEX_-1, Ly108^+^CD69^–^ T_PEX_-2, Ly108^–^CD69^–^ T_EX_ intermediate and Ly108^–^CD69^+^ terminal T_EX_ cells in TILs by the T_PEX_ and T_EX_ markers Ly108 and CD69 in flow cytometry assays. Statistical analysis in **(C, F, H, J)** was performed using unpaired two-tailed Student’s *t*-test with *Welch*’s correction: *p≤ 0.05; **p<0.01; ***p<0.001; ****p<0.0001.

To test the anti-tumor activity of NFATc1 and NFATc2 *in vivo*, we adoptively transferred CD8^+^ T cells deficient for NFATc1 or NFATc2, or for the *Nfatc1* E2 enhancer, into tumor-bearing mice. To this end, 5x10^6^ OT-I T cells were injected into the tail vein of *Rag1^-/-^* mice with established B16/OVA melanomas (**Supplementary Figure 8A**), and tumor progression was monitored over time. Strikingly, mice receiving NFATc2-deficient OT-I T cells showed a marked delay in tumor progression, whereas no decrease in tumor size was observed for NFATc1-deficient and ΔE2 CD8^+^ T cells compared to WT CD8^+^ T cells (**Figures 7A–C**). These findings suggest that ablation of NFATc2 enhances the anti-tumor activity of antigen-specific T cells, likely by rendering them resistant to terminal differentiation and exhaustion-induced dysfunction.

Six days after the adoptive transfer, tumor-infiltrating lymphocytes (TILs) were isolated from tumors and analyzed by flow cytometry. Due to the poor survival of NFATc1-deficient cells, only WT and NFATc2-deficient T cells were recovered in sufficient numbers for further examinations, with NFATc2-deficient OT-I T cells isolated in even higher numbers than their WT counterparts (**Figure 7D**). While both frequency and number of CD44^+^PD-1^+^ TILs were enhanced, the expression of PD-1, TIM-3, and CD69 per cell was markedly reduced in NFATc2-deficient TILs compared to WT controls (**Supplementary Figures 8B–E**). Emphasizing limited exhaustion upon loss of NFATc2, the relative and absolute numbers of CD44^+^TIM-3^+^CD8^+^ T cells decreased significantly (**Supplementary Figure 8D**). In contrast, not only the percentage of CD44^+^Ly108^+^ cells and their expression level of Ly108, but also frequencies of CD44^+^CD62L^+^ central memory T cells were increased in NFATc2-deficient CD8^+^ TILs at the expense of short lived CD44^+^CD62L^–^ effector T cells (**Supplementary Figure 8F**; **Figures 7E, F**). Moreover, NFATc2-deficient TILs were predominantly Ly108^+^TIM-3^-^ T_PEX_ cells (**Figures 7G, H**). Additionally, within the CD44^+^ activated TIL population, NFATc2-deficient T cells contained a higher fraction of Ly108^+^CD69^-^ T_PEX_ cells, and a reduced proportion of Ly108^-^CD69^+^ T_EX_ cells were observed within the NFATc2-deficient population (**Figures 7I, J**), consistent with our findings from chronic *in vitro* stimulation and persistent LCMV infection. Thus, in the tumor setting, adoptively transferred NFATc2-deficient CD8^+^ T cells outperformed both NFATc1-deficient and WT counterparts by resisting the acquisition of a terminally exhausted phenotype, thereby sustaining superior anti-tumor activity and highlighting their potential for therapeutic exploitation.

## Discussion

We present here a novel view on the role of NFATc1 *versus* NFATc2 in the exhaustion of CD8^+^ T cells upon chronic viral infection and tumor challenge. While we confirm that persistent antigenic stimulation of CD8^+^ T cells results in the induction of NFATc1/αA, a short NFATc1 isoform, we reveal that rather than driving exhaustion, NFATc1/αA is essential for the survival of exhausted CD8^+^ T cells. Although NFATc1 may be directly involved in the exhaustion program (22), its deficiency would be compensated by upregulated NFATc2. Indeed, the observed low levels of NFATc2 in T_EX_ cells appear to be mediated by NFATc1/αA, as chronically activated NFATc1- and NFATc1/αA-deficient CD8^+^ T cells allowed normal NFATc2 expression. This is of particular interest, as NFATc2 dampens the activity of CD8^+^ T cells to control chronic LCMV infections and melanoma cells. In contrast, elevated levels of NFATc1/αA not only support the survival of chronically stimulated CD8^+^ T cells but also maintain these cells in a T_PEX_ state by repressing NFATc2 expression. These findings provide a rationale for the therapeutic manipulation of CD8^+^ T cells in immunotherapy. Specifically, our results suggest that the use of NFATc2-deficient CD8^+^ T cells (or of T cells with a dampened expression of NFATc2, e.g. by over-expressing NFATc1/αA) may enhance anti-tumor activity.

Our initial overexpression studies had already suggested that, whereas NFATc2 and the long isoforms of NFATc1 promote the apoptosis of primary T cells, NFATc1/αA has a pro-survival effect (48). Consistent with this, NFATc1/αA has been reported to support the survival of T-follicular helper (T_FH_) cells (56). Notably, predominant NFATc1/αA expression is also a hallmark of CD4^+^ T_FH_ cells (57), which are characterized by hypo-responsiveness and share features with the gene expression signature of the self-renewing CD8^+^ TCF-1^+^ T_PEX_ cells (58, 59). Due to the high level of AP-1-sequestering Bcl-6, NFATc1/αA functions AP-1-independently in T_FH_ cells, promoting TOX family expression, key regulators for T_FH_-cell development, anergy and exhaustion (60, 61). In the context of T-cell exhaustion, the pleiotropy of inhibitory receptors, above all PD-1, interferes with costimulatory signals required for NF-κB and AP-1 activation. Accordingly, previous studies on the role of NFATs in CD8^+^ T-cell exhaustion concluded that “the transcription factor NFAT promotes exhaustion of activated CD8^+^ T cells, if NFATs do not interact with AP-1” (4). Here, we further specify that, in the context of T_PEX_ cell survival, this function is mediated by NFATc1/αA. This concept may not be limited to T cells, as indolent (anergic) B cells in chronic lymphocytic leukemia (CLL) exhibit elevated NFATc1 levels, expressed as NFATc1/αA in a murine CLL model, mediating anergy in an AP-1 independent mode (62).

Interestingly, NFATc1 deficiency shifts the balance between Bim and Bcl-2, which is a critical rheostat for programmed cell death (63). In the context of cancer and persistent viral infections, Bim activity governs whether overstimulated CD8^+^ T cells undergo apoptosis or adapt into a T_PEX/EX_ state. A high Bim/Bcl-2 ratio indicates that the cell’s survival threshold is broken, as excess Bim is freed to bind and neutralize pro-survival proteins, triggering mitochondrial outer membrane permeabilization and subsequent cell death (64). Our data suggest that NFATc1/αA is essential for repressing high levels of Bim, presumably in conjunction with BATF and IRF4 (22, 65).

In chronic infections and cancer, sustained antigen receptor engagement and calcium signaling promotes and prolongs nuclear translocation of NFATs, thereby driving the expression of exhaustion markers. Conversely, inhibiting calcium signaling prevents exhaustion (66). Additionally, we have shown that NFATc1 can be activated via an alternative pathway, initiated by IL-7-mediated tyrosine (Tyr371) phosphorylation. In pre-TCR^–^ thymocytes phosphorylation at Tyr371 leads to robust nuclear translocation of NFATc1, thereby supporting their survival (67, 68). Intriguingly, IL-7 is a key cytokine preventing terminal differentiation to CD8^+^ T_EX_ cells and promoting long-lived memory and T_PEX_ survival (69). Whether tyrosine phosphorylation of NFATc1 occurs in T_PEX_ cells has not been investigated. However, such a mechanism could support the expression of NFATc1/αA.

It is puzzling that our transcriptomic analysis of chronically stimulated CD8^+^ T cells revealed no evidence of a pronounced NFATc1-specific transcriptional program in WT *versus* NFATc1(αA)-deficient cells. NFATc1/αA lacks the C-terminal peptide consisting of approximately 250 amino acid residues, which is typical of most NFAT proteins. This peptide harbors two sequence motifs for SUMOylation (70). We found that avoiding SUMOylation, which results in the sole expression of NFATc1/αA-like NFATc1, fine-tunes T-cell responses in the long term, despite having only transient direct effects on transcription (71). This may be similarly regulated in CD8^+^ T-cell exhaustion.

Our data confirm and expand upon the critical role of NFATc2 in driving CD8^+^ T-cell exhaustion. The absence of NFATc2 in chronically stimulated CD8^+^ T cells resulted in pronounced alterations in RNA and protein expression, supporting the concept that many T_EX_ genes are NFAT target genes (4, 44). The two high-mobility group TFs TOX and TOX2 as well as the three members of the NR4A nuclear receptor family, NR4A1, NR4A2 and NR4A3, are strongly induced by NFATs in exhausted CD8^+^ T cells and control the hypo-responsive state of T_EX_ cells (72, 73). NFAT, TOX and NR4A members, form enhanceosome complexes (74) at the control regions of T_EX_ genes to support exhaustion (72). When NFATc1 is involved in chronic activation of CD8^+^ T cells, it cooperates with BATF and IRF4 (22). Overall, NFATc1 and NFATc2 are known to engage both shared and distinct protein interaction partners, frequently involving other transcription factors (75). While this has not yet been systematically examined in the context of T-cell exhaustion, it may represent a potential mechanism underlying functional differences between NFATc1 and NFATc2 in this setting.

NFATc2 expression was almost lost in terminally differentiated CD8^+^ T_EX_ cells but preserved in NFATc1-deficient cells. This suggests a direct repression by NFATc1/αA. However, the effect on *Nfatc2* RNA was modest, consistent with negligible binding of NFATc1 or NFATc2 to the *Nfatc2* locus (4, 44). Thus, NFATc1-mediated repression of NFATc2 is likely to be (i) indirect and (ii) not necessarily exerted at the transcriptional level.

A study on the death-associated protein-like 1 (Dapl1) (52) offers an explanation for the selective loss of nuclear NFATc2 in terminal T_EX_ cells. Dapl1 promotes CD8^+^ T-cell exhaustion, as its ablation prevents terminal T_EX_ differentiation. The authors confirm that NFATc2 activation is markedly attenuated in CD8^+^ T_EX_ cells, but not in TIM-3^–^ progenitor cells. Upon expression of the inhibitory receptor TIM-3 (4, 44), a negative feedback mechanism targeting NFATc2 is established. This effect is mediated by Dapl1, which selectively binds dephosphorylated NFATc2 – but not NFATc1 – and promotes its rephosphorylation, thereby preventing nuclear translocation. Notably, both NFATc1 ablation and Dapl1 deficiency restore NFATc2 expression in CD8^+^ T_EX_ cells. At the same time, our data show that chronic stimulation in the absence of NFATc2 induces Dapl1 expression, consistent with its predominant expression in T_PEX_ cells (52). However, in the absence of NFATc2, Dapl1 may not be able to promote terminal T-cell exhaustion. Together, further studies are required to elucidate the underlying regulatory network linking Dapl1, NFATc1 *versus* NFATc2, and T-cell exhaustion.

Collectively, we uncovered distinct roles for NFATc1 and NFATc2 in CD8^+^ T-cell responses during chronic antigenic stimulation. While NFATc1 – particularly its inducible isoform NFATc1/αA – supports the survival of progenitor and exhausted T cells, NFATc2 promotes the terminal differentiation and dysfunction of T_EX_ cells. Importantly, ablation of NFATc2 preserves progenitor-like characteristics and enhances the anti-tumor efficacy of adoptively transferred CD8^+^ T cells. These findings position NFATc2 as a potential therapeutic target for enhancing CAR T-cell functionality.

## Data Availability

The datasets presented in this study can be found in online repositories. The names of the repository/repositories and accession number(s) can be found below: https://www.ncbi.nlm.nih.gov/geo/, GSE253492.
