# Supplementary material for: Non-redundant functions of NFATc1 in survival and NFATc2 in generation of exhausted CD8+ T cells
Source: Front Immunol. 2026 Jul 24;17:1869029. doi: 10.3389/fimmu.2026.1869029 (PMC13447309; doi:10.3389/fimmu.2026.1869029)
Supplement: Supplementary file 1 [file DataSheet1.pdf]

Supplementary Material

Non-redundant Functions of NFATc1 in Survival and NFATc2 in Generation of Exhausted CD8⁺ T Cells

Salvador Sampere-Birlanga[1†], Stefan Klein-Hessling[1†], Miriam Campillo Prados[2†], Anfei Huang[2], Hao Wu[2&], Andreas Rosenwald[3], Wolfgang Kastenmüller[2], Edgar Serfling[1§], Martin Vaeth[2,4§], and Friederike Berberich-Siebelt[3§]*

[1] Department of Molecular Pathology, Institute of Pathology, Julius-Maximilians-University of Würzburg, 97080 Würzburg, Germany
[2] Würzburg Institute of Systems Immunology, Max Planck Research Group at the Julius-Maximilians University of Würzburg, 97080 Würzburg, Germany
[3] Institute of Pathology, Julius-Maximilians University of Würzburg, 97080 Würzburg, Germany
[4] Mildred Scheel Early Career Center (MSNZ) of the German Cancer Aid, University Hospital, Julius-Maximilians University of Würzburg, Würzburg, Germany

[†] These three authors have contributed equally to this work and share the first authorship.
[§] These three authors have contributed equally to this work and share the senior authorship.
[&] Present address: Department of Gastroenterology, Gastrointestinal Oncology and Endocrinology, University Medical Center Göttingen, 37075 Göttingen, Germany

*** Correspondence:**
Friederike Berberich-Siebelt
path230@mail.uni-wuerzburg.de
Keywords: CD8⁺ T cells, NFATc1, NFATc2, T-cell exhaustion, T-cell survival

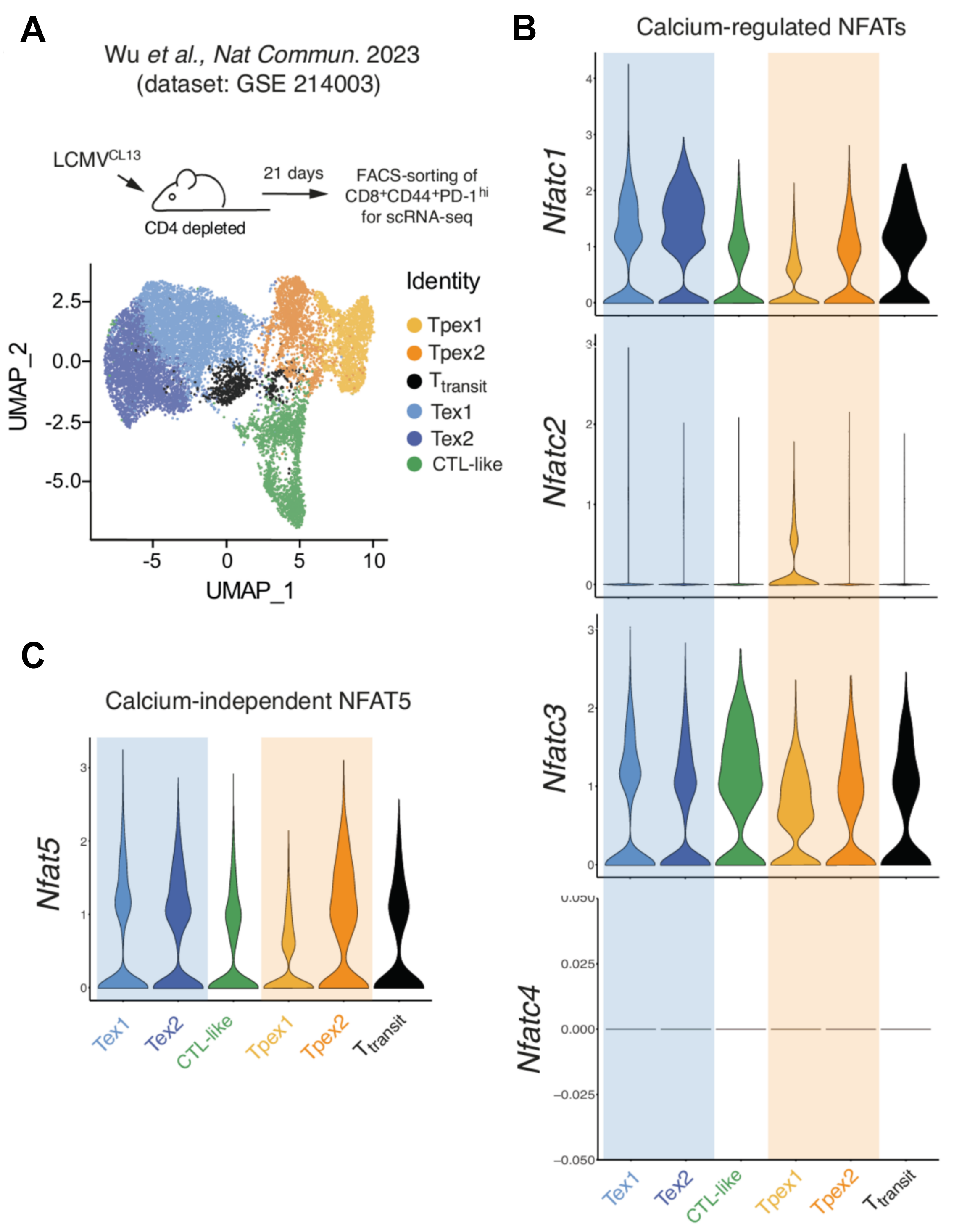

Supplementary Figure S1. Gene expression analysis of different NFAT family members in exhausted T cell subsets from chronically infected WT mice. (**A**) Naïve C57BL/6 mice were chronically infected with the LCMV strain clone 13 (LCMVCL13) and $CD8^{+}CD44^{+}PD\text{-}1^{hi}$ T cells were FACS-sorted and subjected to single-cell RNA sequencing (scRNA-seq) at day 21 post infection. scRNA-seq data was previously published in Wu et al., Nat Commun. 2023 (PMID: 37891230; dataset: GSE214003). (**B**) Violin plots displaying gene expression of calcium-regulated *Nfatc1*, *Nfatc2*, *Nfatc3* and *Nfatc4* in T_{PEX} and T_{EX} cells analyzed by scRNA-seq. (**C**) Violin plots displaying gene expression of calcium-independent *Nfat5* in T_{PEX} and T_{EX} cells analyzed by scRNA-seq.

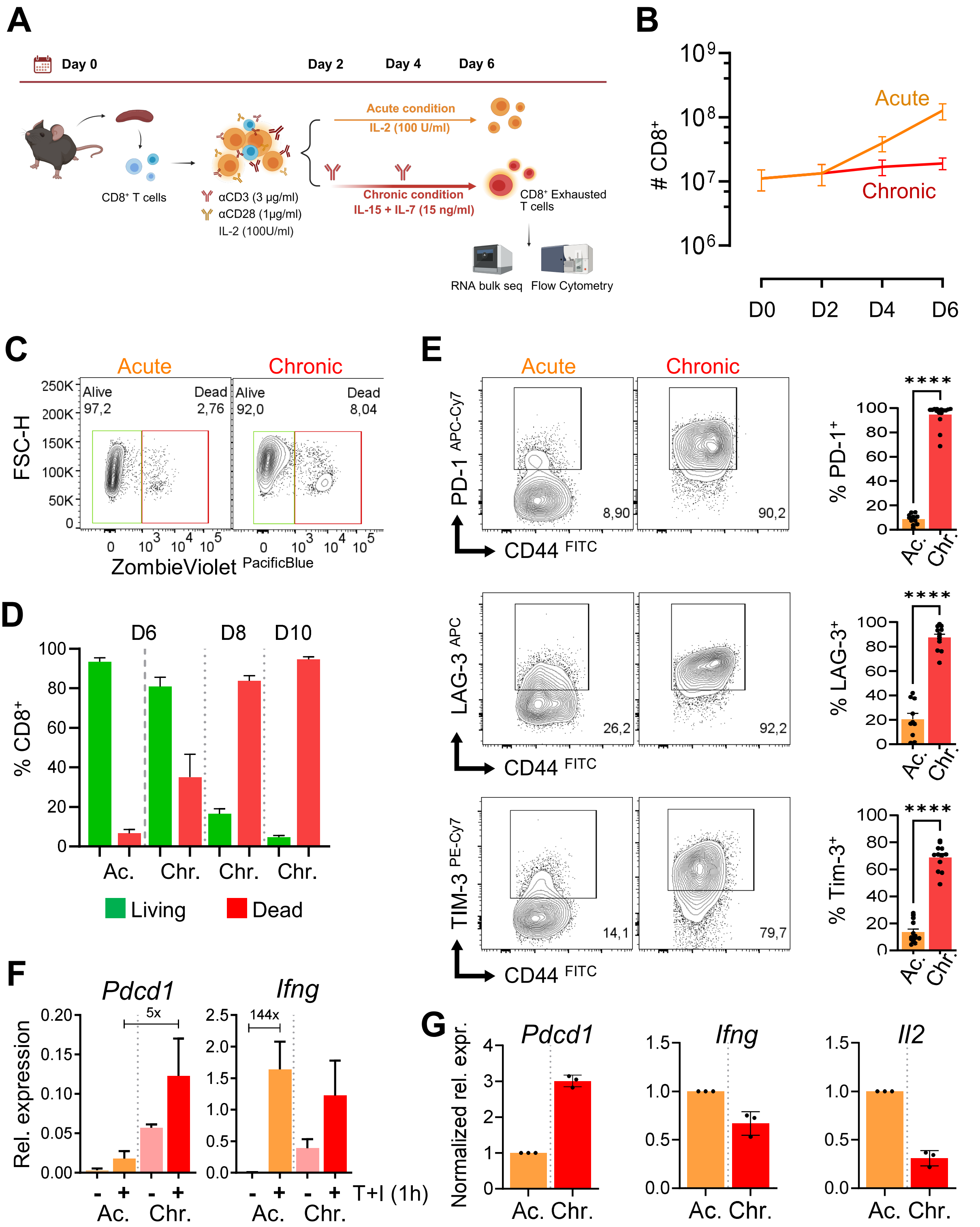

Supplementary Figure S2. Generation of exhausted CD8$^+$ T cells *in vitro*. (**A**) Scheme of *in vitro* generation of exhausted murine CD8$^+$ T cells. (**B**) Cell number of CD8$^+$ T cells over time upon acute (orange) and chronic (red) stimulation for 6 days, n=5; mean ± SEM. (**C**) Representative dot plots showing the viability of CD8$^+$ T cells under acute and chronic conditions at d6, measured by Zombie Violet^{TM} staining of dead cells. (**D**) Quantitation of live (green) and dead (red) CD8$^+$ T cells upon chronic activation for 6, 8 and 10 days; n=5; mean ± SEM. (**E**) Representative dot plots of flow cytometry and quantitation revealing the frequency of PD-1$^+$, LAG-3$^+$, and TIM-3$^+$ CD8$^+$ T cells upon acute (orange) and chronic (red) stimulation for 6 days. n=14-17; unpaired *t*-test with Welch's correction: **** $p<0.0001$. (**F**) Comparative expression levels of *Pdcd1* and *Ifng* RNAs in cells of acute and chronic stimulation, treated by TPA and ionomycin (T+I) during the last hour of culture. qRT-PCRs were performed in 3 independent experiments analyzed each in triplicates, mean ±SEM. (**G**) Fold expression levels of *Pdcd1*, *Ifng*, and *Il2* RNAs in cells of acute and chronic stimulation, treated by TPA and ionomycin (T+I) during the last hour of culture. Data are normalized to acute condition, mean ±SEM.

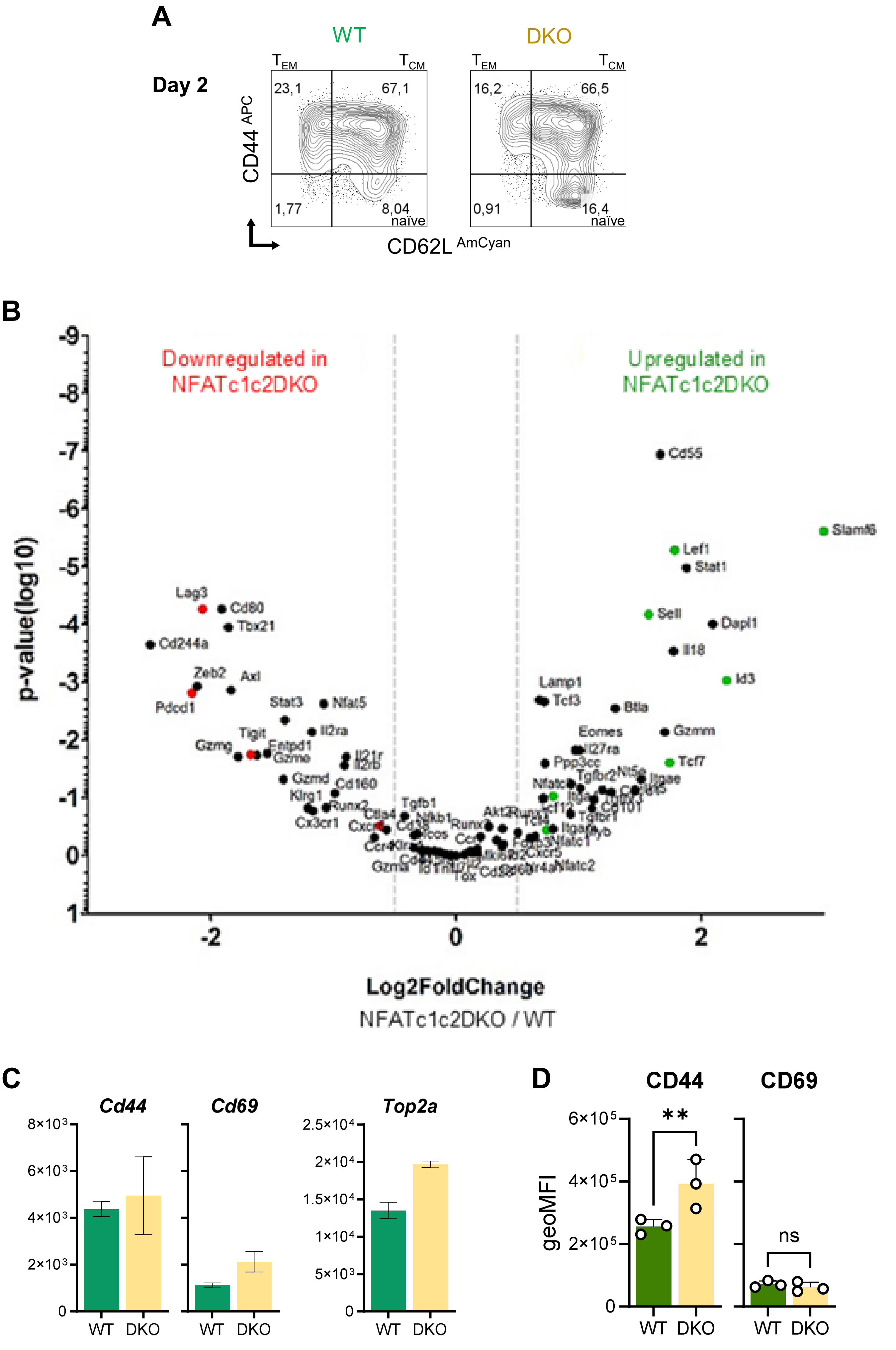

A
Day 2
WT
DKO
T EM
T CM
23,1
67,1
1,77
8,04
naïve
16,2
66,5
0,91
16,4
CD44 APC
CD62L AmCyan
B
Downregulated in NFATc1c2DKO
Upregulated in NFATc1c2DKO
p-value(log10)
Log2FoldChange
NFATc1c2DKO / WT
Cd55
Slamf6
Lef1
Stat1
Sell
Dapl1
Il18
Id3
Lag3
Cd80
Tbx21
Cd244a
Zeb2
Axl
Pdcd1
Stat3
Nfat5
Il2ra
Tigit
Gzmg
Entpd1
Gzme
Il21r
Il2rb
Gzmd
Cd160
Klrg1
Runx2
Cx3cr1
Lamp1
Tcf3
Btla
Eomes
Gzmm
Il27ra
Tcf7
Ppp3cc
Tgfbr2
Itgae
Tgfb1
Nfkb1
Cd101
Tgfbr1
Nfatc1
Nfatc2
Tox
C
Cd44
Cd69
Top2a
8×10³
6×10³
4×10³
2×10³
0
2.5×10⁴
2×10⁴
1.5×10⁴
1×10⁴
5×10³
WT
DKO
D
CD44
CD69
geoMFI
6×10⁵
4×10⁵
2×10⁵
**
ns

Supplementary Figure S3. NFATc1/NFATc2 double deficiency leads to reduced expression of exhaustion-associated genes. (**A**) Representative dot plots showing the memory profile of WT vs. NFATc1c2 double-deficient (DKO) $CD8^+$ T cells at day 2 after primary stimulation. (**B**) Volcano plot showing the results of bulk NGS assays from $CD8^+$ T cells, double-deficient for NFATc1 and NFATc2 (NFATc1c2DKO) compared to WT upon chronic activation for 6 days. The X-axis indicates the log2-fold change expression, and the Y-axis represents the adjusted significance value (–log10 adjusted p-value). T-cell exhaustion relevant (including those for T_{EX} vs. T_{PEX}) transcripts are highlighted in green (upregulated in DKO) or red (downregulated in DKO). (**C**) Individual DESeq2 normalized counts derived from RNA-seq analysis, selected genes that indicate activation and cell cycle. (**D**) Expression of CD44 and CD69 per cell in chronically stimulated WT *versus* DKO $CD8^+$ T cells (geoMFI); unpaired two-tailed Student's *t*-test with *Welch*'s correction: **, $p<0.01$.

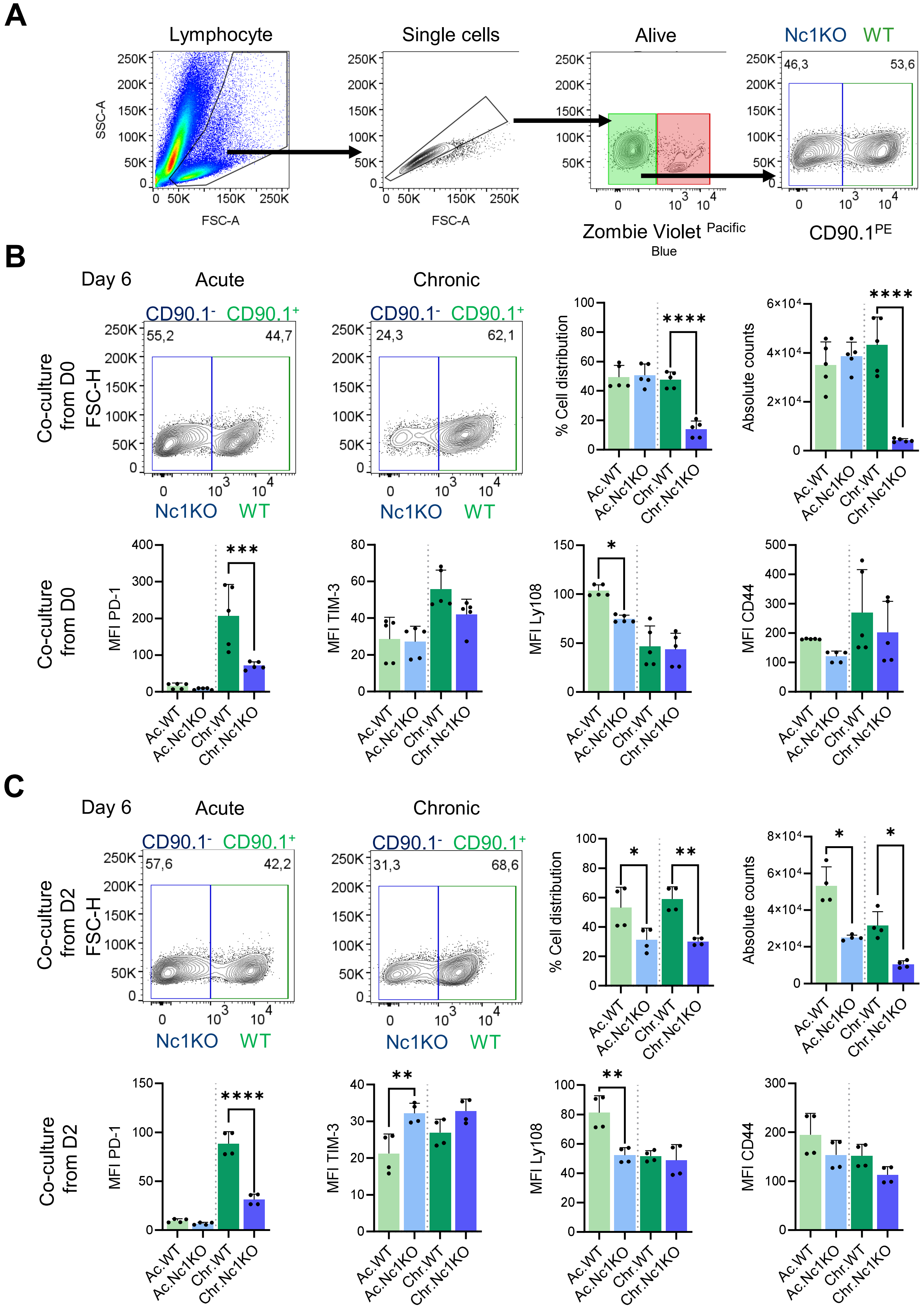
A
Lymphocyte
Single cells
Alive
Nc1KO WT
SSC-A
FSC-A
Zombie Violet Pacific Blue
CD90.1PE
46,3
53,6
250K
200K
150K
100K
50K
0
50K
150K
250K
B
Day 6
Acute
Chronic
Co-culture from D0
CD90.1- CD90.1+
FSC-H
55,2
44,7
24,3
62,1
Nc1KO WT
% Cell distribution
Absolute counts
6×10⁴
4×10⁴
2×10⁴
Ac.WT
Ac.Nc1KO
Chr.WT
Chr.Nc1KO
MFI PD-1
MFI TIM-3
MFI Ly108
MFI CD44
****
***
*
C
Co-culture from D2
57,6
42,2
31,3
68,6
8×10⁴
**

Supplementary Figure S4. Rapid loss of NFATc1-deficient CD8⁺ T cells in co-cultures with WT CD8⁺ T cells during chronic stimulation. (**A**) Scheme of flow cytometry strategy. (**B**) Co-incubation assays. $5x10^5$ CD90.1⁺ WT- and CD90.1⁻ (CD90.2⁺) NFATc1-deficient T cells were mixed, co-stimulated for 2 days and co-incubated for 4 days under acute and chronic conditions (see Figure 1A). At d6, the composition of cultures was determined by flow cytometry. The column presentations show the distribution and absolute numbers of cells from 3 assays after co-incubation. In addition, they show the expression levels of PD-1, Ly108, CD69, TIM-3 and CD44 markers from 3 co-incubations. (**C**) CD90.1⁺ WT- and CD90.1⁻ (CD90.2⁺) NFATc1-deficient T cells were stimulated for 2 days in separated vials followed by co-incubation for 4 days (and flow cytometry as in (B)). Statistical analysis in (B+C) was performed using unpaired two-tailed Student's *t*-test with *Welch*'s correction: *, p≤0.05, **, p<0.01; ***, p<0.001; **** p<0.0001.

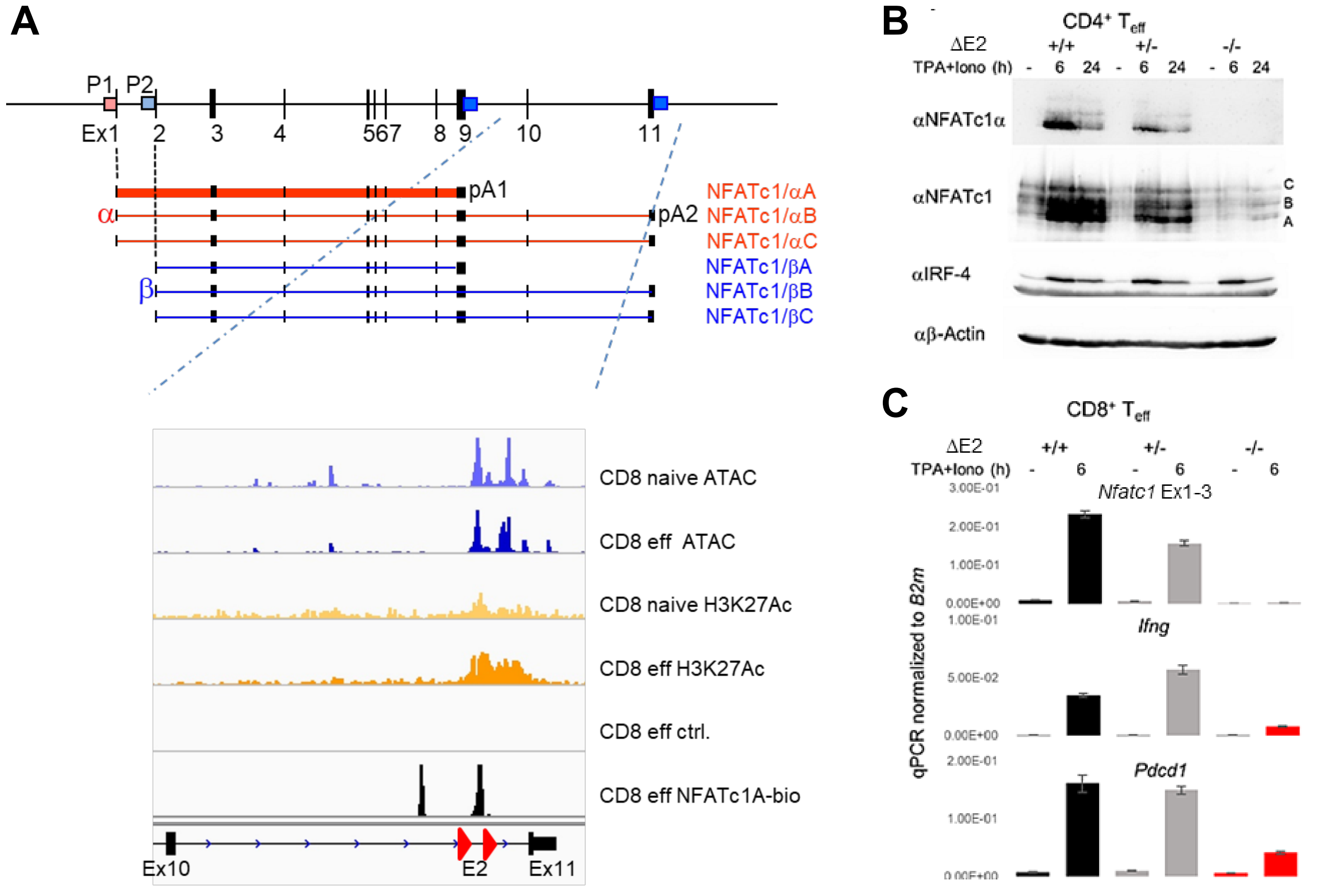

Supplementary Figure S5. Deletion of the E2 enhancer abolishes NFATc1/αA induction in T cells. (**A**) Scheme of the murine *Nfatc1* gene, with the inducible promoter P1 (soft red) and the constitutive P2 (soft blue), the localization of the E2 enhancer in intron10 (loxP sites as red triangles), the position of chromatin modifications and binding of NFATc1, leading to the expression of three P1-dependent exon1-encoded α-peptide-containing isoforms (including the dominant NFATc1/αA) in red and three P2-dependent exon2-encoded β-peptide-containing isoforms in blue, modified from Rudolf et al., 2014; 10.3389/fimmu.2014.00021. (**B**) Immunoblots of proteins from CD4+ T-effector cells induced by TPA+ionomycin *in vitro* from WT mice (+/+) or mice bearing a heterozygous (+/-) or homozygous deletion (-/-) of E2 showing the ablation of NFATc1/αA, -αB, and -αC induction in CD4+ effector T cells. The uppermost blot was performed with an Ab specific for the α-peptide of NFATc1, the other blots with Abs recognizing all NFATc1 proteins, IRF-4 or β-actin. Note that E2 deletion does not affect the basic expression of NFATc1, but it does abolish all inducible expression. (**C**) Results of qPCR assays showing the effect of E2 deletion on the RNA induction of *Nfatc1* (using exon1-exon3 specific primers), *Ifng* and *Pdcd1* genes in CD8+ T-effector cells upon TPA+ionomycin induction *in vitro*.

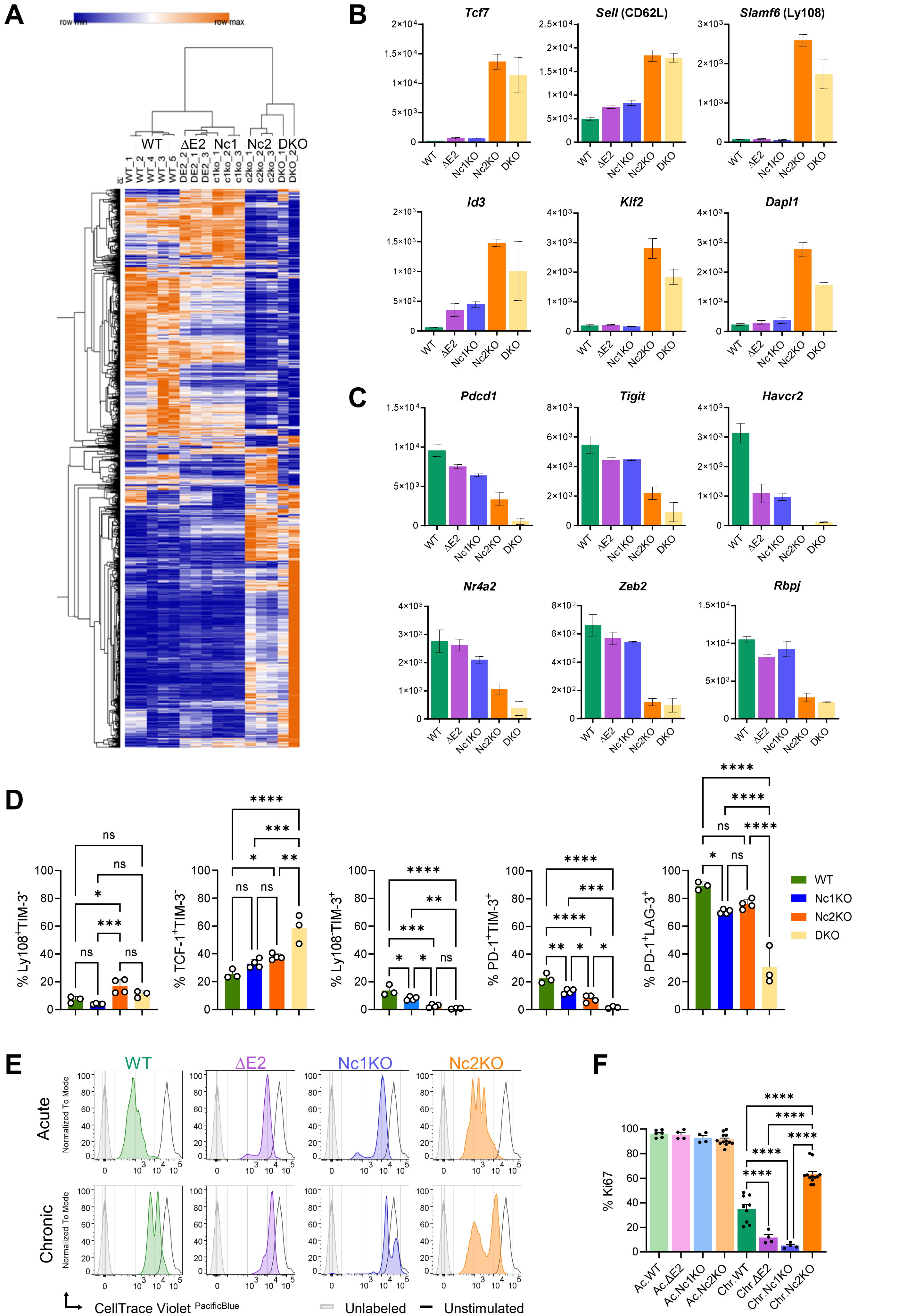
A
row min
row max
WT
ΔE2
Nc1
Nc2
DKO
B
Tcf7
Sell (CD62L)
Slamf6 (Ly108)
Id3
Klf2
Dapl1
C
Pdcd1
Tigit
Havcr2
Nr4a2
Zeb2
Rbpj
WT
ΔE2
Nc1KO
Nc2KO
DKO
D
% Ly108+TIM-3-
% TCF-1+TIM-3-
% Ly108-TIM-3+
% PD-1+TIM-3+
% PD-1+LAG-3+
WT
Nc1KO
Nc2KO
DKO
E
WT
ΔE2
Nc1KO
Nc2KO
Acute
Chronic
Normalized To Mode
CellTrace Violet PacificBlue
Unlabeled
Unstimulated
F
% Ki67
Ac.WT
Ac.ΔE2
Ac.Nc1KO
Ac.Nc2KO
Chr.WT
Chr.ΔE2
Chr.Nc1KO
Chr.Nc2KO

Supplementary Figure S6. Gene expression profiling of chronically stimulated NFAT-deficient CD8$^+$ T cells. (**A**) Heat map of all 1766 genes that were differently expressed (base mean >100, p<0,05, FC>2) in CD8$^+$ T cells upon chronic stimulation for 6 days. For each lane, splenic CD8$^+$ T cells from two or three mice were pooled and subjected to chronic stimulation and bulk NGS assays. (**B**) Module of selected genes that are predominantly expressed in T_{PEX} cells or upon NFATc2 ablation. (**C**) Module of selected genes that are predominantly expressed in T_{EX} cells but less in NFATc2-deficient cells. (**D**) Frequency of Ly108$^+$TIM-3$^–$ or TCF-1$^+$TIM-3$^–$ T_{PEX} *versus* Ly108$^–$TIM-3$^+$ or PD-1$^+$TIM-3$^+$ or PD-1$^+$LAG-3$^+$ T_{EX} subpopulations measured by flow cytometry, after chronic stimulation for 6 days. Unpaired *t*-test with Welch's correction: *, p<0.05; **, p<0.01; ***, p<0.001; **** p<0.0001. (**E**) Proliferation assay of CD8$^+$ T cells from day 2 to day 6 under acute and chronic protocols by fluorescent dye dilution (CellTrace^{TM} violet). The black line represents unstimulated cells (max MFI peak) and the gray line represents unlabeled cells (min MFI peaks). CD8$^+$ T cells from 6 WT, 4 ΔE2, 4 *Nfatc1*$^{fl/fl}$.CD4-cre, 10 *Nfatc2*$^{–/–}$ mice. (**F**) Frequencies of Ki67$^+$ cells measured by flow cytometry. One-way ANOVA **** p≤0.0001.

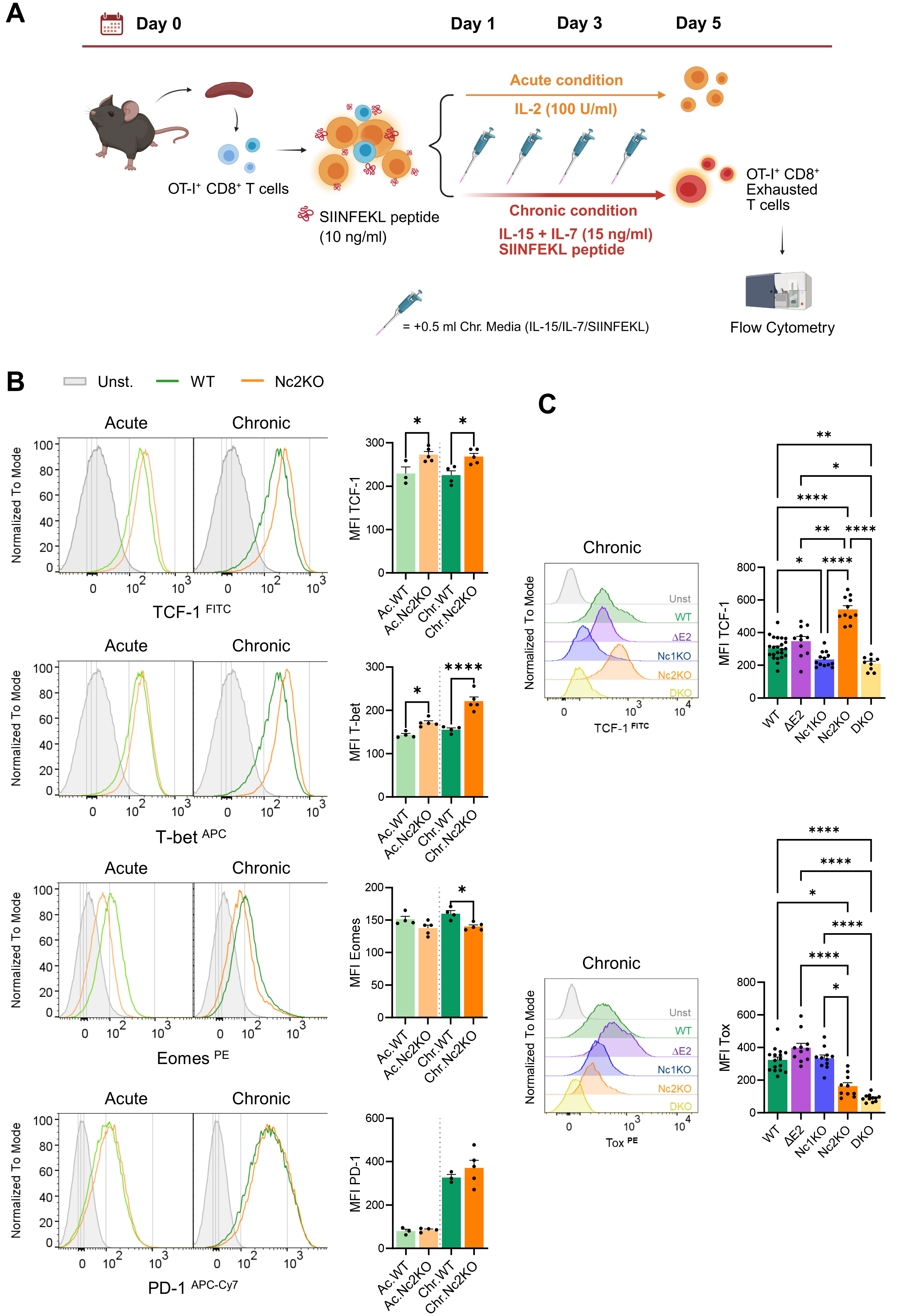
A
Day 0
Day 1
Day 3
Day 5
OT-I⁺ CD8⁺ T cells
SIINFEKL peptide (10 ng/ml)
Acute condition
IL-2 (100 U/ml)
Chronic condition
IL-15 + IL-7 (15 ng/ml)
SIINFEKL peptide
OT-I⁺ CD8⁺ Exhausted T cells
Flow Cytometry
= +0.5 ml Chr. Media (IL-15/IL-7/SIINFEKL)
B
Unst.
WT
Nc2KO
Acute
Chronic
Normalized To Mode
TCF-1 FITC
T-bet APC
Eomes PE
PD-1 APC-Cy7
MFI TCF-1
MFI T-bet
MFI Eomes
MFI PD-1
Ac.WT
Ac.Nc2KO
Chr.WT
Chr.Nc2KO
C
Chronic
Unst
WT
ΔE2
Nc1KO
Nc2KO
DKO
TCF-1 FITC
Tox PE
MFI TCF-1
MFI Tox
WT
ΔE2
Nc1KO
Nc2KO
DKO

Supplementary Figure S7. Chronic stimulation with the OVA-peptide SIINFEKL drives the generation of exhausted OT-I T cells. (**A**) Scheme of the repetitive stimulation of WT and NFATc2-deficient OT-I $CD8^+$ T cells with the Ova-peptide SIINFEKL. After one day, cells were divided into two groups: “acute” in the presence of IL-2 only and “chronic” with daily SIINFEKL and IL-7 + IL-15 additions. All cells were harvested on day 5. (**B**) Flow cytometry of expression of TCF-1, T-bet, Eomes and PD-1 on WT and NFATc2-deficient OT-I $CD8^+$ T cells upon acute stimulation or chronic stimulation with SIINFEKL. (**C**) Flow cytometry of expression levels (MFI) of TCF-1 and TOX in chronically stimulated (protocol in S2A) $CD8^+$ T cells from WT and all NFAT-deficient mice. Statistical analysis was performed using unpaired two-tailed Student’s *t*-test with *Welch*'s correction: *, $p<0.05$; **** $p<0.0001$.

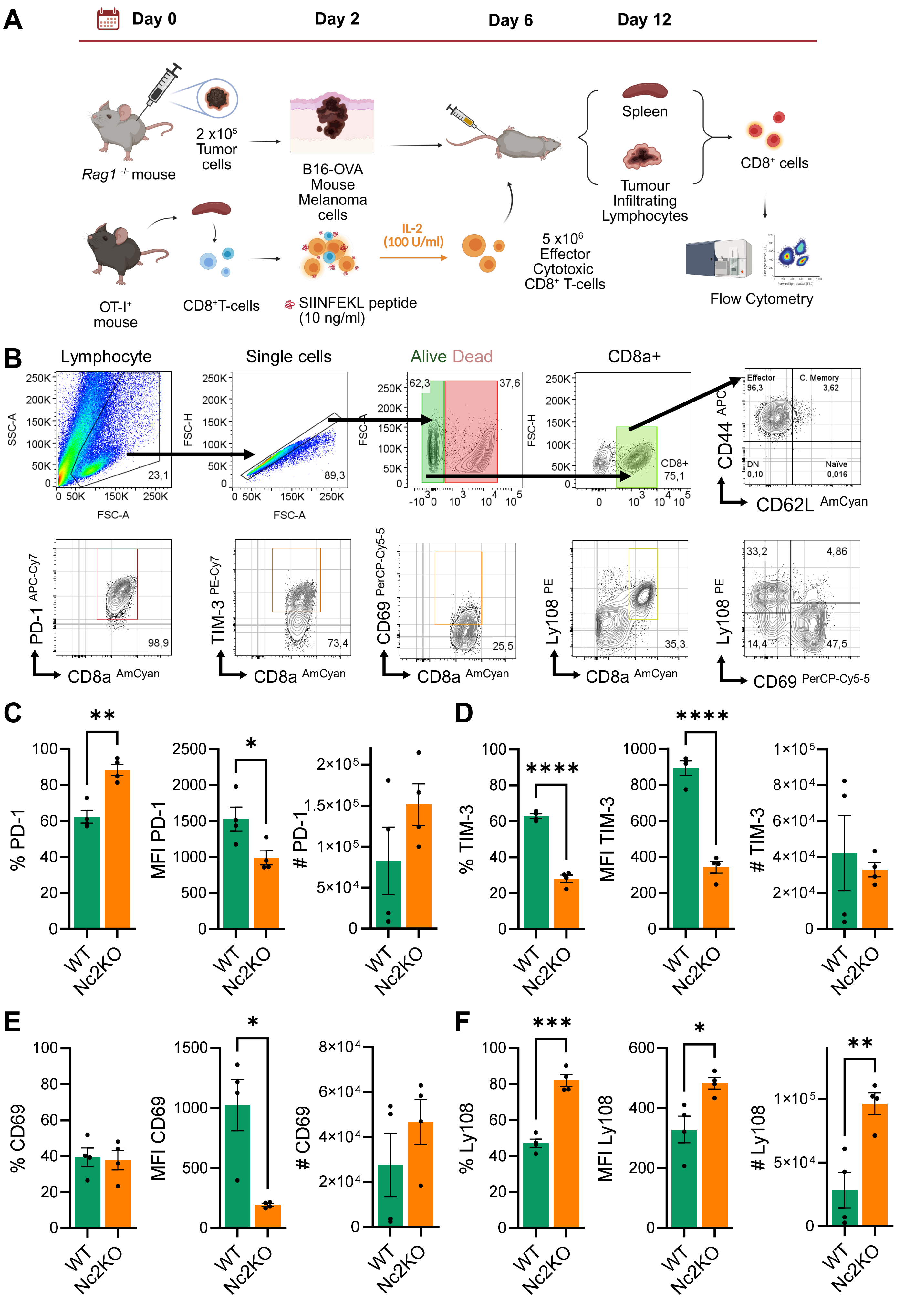

A
Day 0
Day 2
Day 6
Day 12
Rag1 -/- mouse
2 x10^5 Tumor cells
B16-OVA Mouse Melanoma cells
Spleen
Tumour Infiltrating Lymphocytes
CD8+ cells
OT-I+ mouse
CD8+T-cells
SIINFEKL peptide (10 ng/ml)
IL-2 (100 U/ml)
5 x10^6 Effector Cytotoxic CD8+ T-cells
Flow Cytometry
B
Lymphocyte
Single cells
Alive Dead
CD8a+
Effector 96,3
C. Memory 3,62
DN 0,10
Naïve 0,016
CD44 APC
CD62L AmCyan
PD-1 APC-Cy7
TIM-3 PE-Cy7
CD69 PerCP-Cy5-5
Ly108 PE
CD8a AmCyan
CD69 PerCP-Cy5-5
C
D
E
F
% PD-1
MFI PD-1
PD-1
% TIM-3
MFI TIM-3
TIM-3
% CD69
MFI CD69
CD69
% Ly108
MFI Ly108
Ly108
WT
Nc2KO

Supplementary Figure S8. Flow cytometric analysis of WT and NFATc2-deficient TILs. (**A**) Strategy of adoptive transfer of $CD8^+$ T cells into tumor mice, and the isolation and analysis of TILs. $5x10^6$ $CD8^+$ T cells "acutely" pre-stimulated with SIINFEKL were injected into the tail vein of *Rag1*$^{-/-}$ mice bearing B16/Ova melanomas. 12 days later the tumors were harvested, their size was measured, and their TILs were prepared for flow cytometry (n=4). (**B**) Strategy of flow cytometry and identification of marker proteins within the population of $CD8a^+$ TILs. (**C-F**) Quantitation of expression of marker proteins PD-1, TIM-3, CD69, and Ly108 within $CD44^+CD8a^+$ WT *versus Nfatc2*-deficient TILs. The frequency (%), expression level per cell (MFI) and absolute number of cells are presented. Statistical analysis was performed using unpaired two-tailed Student's *t*-test with *Welch's* correction: *, p<0.05; **, p<0.01; ***, p<0.001**;** **** p<0.0001
